# Microglial diversity along the hippocampal longitudinal axis impacts synaptic plasticity in adult male mice under homeostatic conditions

**DOI:** 10.1186/s12974-022-02655-z

**Published:** 2022-12-08

**Authors:** E. De Felice, E. Gonçalves de Andrade, M. T. Golia, F. González Ibáñez, M. Khakpour, M. A. Di Castro, S. Garofalo, E. Di Pietro, C. Benatti, N. Brunello, F. Tascedda, B. Kaminska, C. Limatola, D. Ragozzino, M. E. Tremblay, S. Alboni, L. Maggi

**Affiliations:** 1 Department of Physiology and Pharmacology, Sapienza University of Rome, Piazzale Aldo Moro, 5, 00185 Rome, Italy; 2 Division of Medical Sciences, University of Victoria, Victoria, Canada; 3 Faculté de Médecine and Centre de Recherche, CHU de Québec-Université Laval, Quebec, Canada; 4 Department of Life Sciences, University of Modena and Reggio Emilia, Modena, Italy; 5 Centre of Neuroscience and Neurotechnology, University of Modena and Reggio Emilia, Modena, Italy; 6 Laboratory of Molecular Neurobiology, Nencki Institute of Experimental Biology of the Polish Academy of Sciences, Warsaw, Poland; 7 IRCCS Neuromed, Pozzilli, Italy; 8 Department of Physiology and Pharmacology, Laboratory Affiliated to Istituto Pasteur, Sapienza University, Rome, Italy; 9 Santa Lucia Foundation (IRCCS Fondazione Santa Lucia), Rome, Italy

**Keywords:** LTP, Dorsal hippocampus, Ventral hippocampus, Microglial morphology, Microglial ultrastructure, CX3CL1–CX3CR1 signaling, Cytokines, K^+^ current

## Abstract

The hippocampus is a plastic brain area that shows functional segregation along its longitudinal axis, reflected by a higher level of long-term potentiation (LTP) in the CA1 region of the dorsal hippocampus (DH) compared to the ventral hippocampus (VH), but the mechanisms underlying this difference remain elusive. Numerous studies have highlighted the importance of microglia–neuronal communication in modulating synaptic transmission and hippocampal plasticity, although its role in physiological contexts is still largely unknown. We characterized in depth the features of microglia in the two hippocampal poles and investigated their contribution to CA1 plasticity under physiological conditions. We unveiled the influence of microglia in differentially modulating the amplitude of LTP in the DH and VH, showing that minocycline or PLX5622 treatment reduced LTP amplitude in the DH, while increasing it in the VH. This was recapitulated in Cx3cr1 knockout mice, indicating that microglia have a key role in setting the conditions for plasticity processes in a region-specific manner, and that the CX3CL1–CX3CR1 pathway is a key element in determining the basal level of CA1 LTP in the two regions. The observed LTP differences at the two poles were associated with transcriptional changes in the expression of genes encoding for *Il-1*, *Tnf-α*, *Il-6*, and *Bdnf*, essential players of neuronal plasticity. Furthermore, microglia in the CA1 SR region showed an increase in soma and a more extensive arborization, an increased prevalence of immature lysosomes accompanied by an elevation in mRNA expression of phagocytic markers *Mertk* and *Cd68* and a surge in the expression of microglial outward K^+^ currents in the VH compared to DH, suggesting a distinct basal phenotypic state of microglia across the two hippocampal poles. Overall, we characterized the molecular, morphological, ultrastructural, and functional profile of microglia at the two poles, suggesting that modifications in hippocampal subregions related to different microglial statuses can contribute to dissect the phenotypical aspects of many diseases in which microglia are known to be involved.

**Supplementary Information:**

The online version contains supplementary material available at 10.1186/s12974-022-02655-z.

## Introduction

The hippocampus is an elongated brain structure located in the medial temporal lobe that in rodents runs along a dorsal (septal)-to-ventral (temporal) axis [1, 2]. Far from being structurally homogeneous, the hippocampus shows a functional segregation along its transversal [3, 4] and longitudinal [2] axes. Electrophysiological and lesion studies have suggested a functional discrimination within the hippocampus: the dorsal hippocampus (DH) is involved mainly in cognitive processes linked to spatial memory and navigation, and the ventral hippocampus (VH) to emotional responses like fear and anxiety [2, 5–10]. Recent studies confirmed the co-existence of gradual and discrete transitions along the hippocampal longitudinal axis, depending on the observational level of granularity [2, 11]. The two hippocampal poles differ in their gene expression [12–14], levels of glutamatergic, GABAergic, nicotinic, dopaminergic, and noradrenergic receptors [15], as well as voltage-gated ion channels [15–17]. Interestingly, the *Cornu Ammonis* (CA)1 pyramidal neurons also show different electrophysiological properties along the hippocampal longitudinal axis, such as excitability, short- and long-term plasticity [18–22]. In addition, the amplitude of the long-term potentiation (LTP) is higher in the DH compared to the VH [21, 23, 24]. These differences could be partially explained by ultrastructural variations in CA1 pyramidal cells [18], higher levels of GABAergic inhibition in the VH [21], and the higher expression of small-conductance calcium-activated potassium channels, that suppress *N*-methyl-d-aspartate receptor (NMDAR)-dependent post-synaptic potentials amplification at ventral synapses [25]. In addition, other mechanisms mediated by neuron–glia communications could be involved. Under physiological conditions, microglia, the resident innate immune cells of the brain, were shown to play a central role in modulating learning, memory, as well as neural plasticity and circuit remodeling [26–30].

The vast array of receptors expressed by microglia constantly surveying their surroundings constitutes a ‘sensome’ allowing detection and response to different stimuli arising from sensory and behavioral experiences [31–33]. Moreover, by releasing immune mediators, these cells participate in regulating synaptic transmission and plasticity. For instance, the cytokines interleukin (IL)-1, tumor necrosis factor (TNF)-alpha, and IL-6, expressed at physiologically low levels, are important for the regulation of hippocampal plasticity (i.e., LTP) [34–36]. The crosstalk between neurons and microglia involves communication between ligands and receptors. The best characterized molecular interplay is between the chemokine fractalkine (CX3CL1), mainly found on neurons, and its unique receptor, CX3CR1, predominantly expressed on microglia, which supports the homeostatic role of microglia in learning and memory. Previous data demonstrated that impaired fractalkine signaling in *Cx3cr1* knockout mice potentiated the hippocampal synaptic plasticity in the ventral/central hippocampal regions [37, 38]. Microglia interact with neuronal circuits to mediate several key functional processes such as brain circuit formation, adult hippocampal neurogenesis [39, 40] and activity-dependent synaptic remodeling [41–44]. Dynamic changes in microglial state induced by environmental stimuli and local brain activity have been proposed to affect brain function and consequent behavioral outcomes, thus contributing to experience-dependent plasticity [45].

Although some evidence indicate the relevance of microglial regional heterogeneity in pathological states [46, 47], very few studies have investigated their structural and functional features in the healthy brain and especially along the hippocampal longitudinal axis. To our knowledge, only one paper reported dorsoventral, interregional, and interlaminar differences between microglia of the two hippocampal poles in adult male mice [48]. Specifically, the density of microglia in the VH was significantly lower in the CA3 region than in the CA1 region and dentate gyrus, although no interregional differences were detectable in the DH [48].

In the present study, we examined the contribution of microglia in modulating short- and long-term plasticity in the CA1 region of the DH and VH. To this purpose, we interfered with the physiological functions of microglia using different approaches: (i) a functional modulation with the tetracycline antibiotic minocycline in vitro; (ii) a pharmacological depletion with PLX5622 in vivo, and (iii) a genetic deletion of the *Cx3cr1* gene in a mouse model where neuron–microglia communication is impaired. Moreover, we extensively characterized the molecular, morphological, ultrastructural, and functional features of microglia among the DH versus VH under physiological conditions. We hypothesized that microglia might contribute to determining the distinct plasticity potential at the two hippocampal poles. Regional morphological and physiological adaptations of microglia can affect their interactions with the surrounding and can potentially influence their response to insults in a region-specific manner. Thus, our findings may have important implications for basic, translational, and clinical research pertaining to neurodevelopmental, neuropsychiatric, and neurodegenerative disorders engaging the hippocampus and in which microglia were found to be involved throughout lifespan.

## Methods

### Animals

Experiments described in the present work were approved by the Italian Ministry of Health in accordance with the guidelines on the ethical use of animals from the European Community Council Directive of September 22, 2010 (2010/63/EU), the Italian D. Leg. 26/2014, as well as by Université Laval’s animal ethics committee in accordance with the Canadian Council on Animal Care. All possible efforts were made to minimize animal suffering and to reduce the number of animals used per condition by calculating the necessary sample size before performing the experiments.

Adult (8–12 weeks old) male mice were used: C57BL/6J wild-type (CTRL) and *Cx3cr1*^GFP/GFP^, on a C57BL/6J background (from the Jackson Laboratory, Charles River, where the *Cx3cr1* gene was replaced by a green fluorescent protein—GFP—reporter) [49]. In this work, we refer to these mice as Cx3cr1^−/−^ mice. *Cx3cr1*^+/GFP^ were used for patch-clamp recordings.

Mice were housed in a standard breeding cage at constant temperature (22 ± 1 °C) and relative humidity (50%), under a 12-h light–dark cycle. Food and water were available ad libitum.

### Treatments

Only CTRL mice were subjected to pharmacological treatments. Specifically, to modulate microglial functions, dorsal and ventral hippocampal slices from CTRL animals were pre-treated for 1 h with the tetracycline antibiotic minocycline (MINO) in the incubation chamber, and then continuously superfused in the recording chamber during the electrophysiological assessment, therefore the drug was present for 2 h until the LTP protocol induction [50]. To transiently deplete microglia, a group of mice was treated for 7 consecutive days with PLX5622 (PLX), a selective inhibitor of colony stimulating factor (CSF) 1 receptor, essential for microglial proliferation, differentiation, and survival [51–54]. PLX5622 was kindly provided by Plexxikon Inc. (Berkeley, USA) and formulated in standard chow at 1200 mg/kg by Research Diets. Drug-free standard chow (Research Diets) was used in control experiments and during PLX5622 withdrawal.

### Hippocampal slices preparation

To perform electrophysiological experiments, acute hippocampal slices from CTRL, Cx3cr1^−/−^, and Cx3cr1^+/GFP^ mice were collected. The animals were anesthetized by inhalation of halothane (Merck KGaA, Darmstadt, Germany) and decapitated. The brain was rapidly removed from the skull and immersed for 10 min in ice-cold cutting solution. For field recordings, an artificial cerebrospinal fluid (ACSF) solution containing (in mM): NaCl 125, KCl 4, CaCl_2_ 2.5, MgSO_4_ 1.5, NaH_2_PO_4_ 1, NaHCO_3_ 26 and glucose 10, was used for cutting. For patch-clamp recordings, a sucrose-based solution containing (in mM): NaCl 87, KCl 2, CaCl_2_ 0.5, MgCl_2_ 7, NaH_2_PO_4_ 1.2, NaHCO_3_ 25, glucose 10, and sucrose 75 was used for cutting. Solutions were continuously oxygenated with 95% O_2_ and 5% CO_2_ to maintain a proper pH value of 7.35.

Following removal, the brain was hemisected along the longitudinal fissure to separate the two hemispheres. Brain dissection was carried out according to the slicing plane chosen and the structure to be investigated. Specifically, for experiments examining the VH, slices were cut perpendicular to the longitudinal axis from the temporal pole of the brain. Coronal slices were cut from the frontal pole for experiments on the DH. Dorsal and ventral slices were identified as the distance, in μm, from the frontal and temporal poles, respectively (approximately from 400 to 1750 μm). Dorsal and ventral slices were prepared from separate hemispheres of the same brain and were obtained alternately from the right or left hemisphere. The brain tissues were blocked on the stage of a vibratome (Thermo Scientific, USA), then 350 μm (field) or 250 μm (patch) thick slices were cut in ice-cold solutions.

### Electrophysiological recordings

#### Extracellular field recordings

For field recordings, after 2 h of recovery at 30 °C in an incubation chamber containing oxygenated ACSF, individual slices were transferred to the interface slice-recording chamber (BSC1, Scientific System Design Inc.) where they were maintained at 30–32 °C and constantly superfused with oxygenated ACSF at the rate of 1.5 ml/min. Solutions were applied to the slices using a peristaltic pump (Bio-Rad). Slices were visualized with a Wild M3B (Heerbrugg, Switzerland). Experiments were performed from 1 to 7 h after slicing. At the beginning of each recording, a concentric bipolar stimulating electrode (SNE-100X 50-mm-long Elektronik—Harvard Apparatus GmbH) was placed in the hippocampus CA1 *stratum radiatum* (SR) for stimulation of the Schaffer collateral pathway projections to the CA1. A glass micropipette (0.5–1 MΩ) filled with ACSF was placed in the CA1 hippocampal region, at 200–600 µm from the stimulating electrode, to record orthodromically evoked field extracellular post-synaptic potentials (fEPSP). fEPSPs were recorded and filtered (low pass at 1 kHz) with an Axopatch 200A amplifier (Axon Instruments, CA) and digitized at 10 kHz with an A/D converter (Digidata 1322A, Axon Instruments). Stimuli consisted of 100 μs constant current pulses of variable intensity, applied at 0.05 Hz. In each experiment, stimulus intensity was adjusted to evoke ~ 50% of the maximal fEPSP amplitude without appreciable population spike contamination. Evoked responses were monitored online and stable baseline responses (variation in the amplitude values under 10%) were recorded for at least 10 min. Only slices showing stable fEPSP amplitudes were included in the experiments. LTP was induced by high-frequency stimulation (HFS, 2 trains of stimuli at 100 Hz of 1 s duration each, 3 s inter-train interval). To analyze the time course of fEPSP amplitude, the recorded fEPSP was routinely averaged over 1 min (*n* = 3 traces). fEPSP amplitude changes following the LTP induction protocol were calculated with respect to the baseline (35 min after versus 1 min before LTP induction). The paired-pulse ratio (PPR) was measured from responses to two synaptic stimuli at 50 ms inter-stimulus interval. The PPR was calculated at baseline as the ratio between the fEPSP amplitude evoked by the second stimulus (A2) over the first (A1; A2/A1).

Data were stored on a computer using pClamp 10 software (Axon Instruments) and analyzed offline with Clampfit 10 program (Axon Instruments).

#### Patch-clamp recordings

For recordings of voltage-dependent potassium current from microglia, slices were prepared from Cx3cr1^+/GFP^ mice. After 1 h of recovery at room temperature, slices were transferred to a recording chamber, superfused with an ACSF containing (in mM) NaCl 125, KCl 2, CaCl_2_ 2, MgCl_2_ 1.2, NaH_2_PO_4_ 1.2, NaHCO_3_ 25, and glucose 10, at a rate of approximately 2 ml/min with a gravity-driven perfusion system. GFP-positive cells were visualized using an upright microscope (Leica DM-LFS) equipped with a water immersion 40× objective (Leica) and a digital DCC camera (C8484, Hamamatsu). GFP-expressing microglial cells were visually identified under epifluorescence (Leica EL6000). Fluorescent cells were patched in whole-cell configuration in the CA1 (SR). Micropipettes (4–5 MΩ) were usually filled with a solution containing the following composition (in mM): KCl 135, BAPTA 5, MgCl_2_ 2, HEPES 10, and Mg-ATP 2 (pH 7.35 adjusted with KOH, osmolarity 290 mOsm). Voltage-clamp recordings were performed using a Multiclamp 700B amplifier (Molecular Devices). Currents were filtered at 2 kHz, digitized (10 kHz) and collected using Clampex 10 (Molecular Devices); the analysis was performed offline using Clampfit 10 (Molecular Devices). Since the slicing procedure might cause a response from microglia, especially near the surface of the slices, recordings were performed on cells located in a deeper layer. The current/voltage (*I*/*V*) relationship was determined by applying voltage steps from − 150 to + 70 mV (ΔV = 10 mV) of 200 ms duration, holding the cells at − 70 mV. Resting membrane potential was measured in current clamp mode, at the start of the experiment. Membrane capacitance was estimated as the total charge (i.e., the current integral, Qstep) mobilized in each cell by a 10-mV depolarizing step (Vstep): Qstep/Vstep. To determine K^+^ current densities of individual cells, K^+^ current peak amplitudes were normalized to the cell capacitance. The input resistance, Rin, was measured using the same protocol at the end of a 30-ms pulse, when the current trace reached the steady-state. Outward and inward rectifier K^+^ currents (K_or_ and K_ir_, respectively) amplitudes were evaluated after subtracting the leak current (P/N 4, CLAMPEX, molecular devices). Cells were considered to express the outward rectifier K^+^ current when the *I*/*V* relationship showed a rectification above − 30 mV and the amplitude measured at 0 mV was at least 5 pA, after leak subtraction.

### Light and electron microscopy

For light and scanning electron microscopy (SEM) analyses, CTRL mice were anesthetized with a mix of ketamine (80 mg/kg)/xylazine (10 mg/kg) and transcardially perfused with phosphate-buffered saline (PBS; 50 mM, pH7.4), followed by 3.5% acrolein and 4% paraformaldehyde. Coronal brain sections with 50 μm thickness were cut in ice-cold PBS using a vibratome (VT1200S, Leica Biosystems) and stored at − 20 °C in cryoprotectant (30% (v/v) glycerol and 30% (v/v) ethylene glycol in PBS) until further processing.

#### IBA1 immunoperoxidase staining

Sections containing the DH (Bregma − 1.55 mm to − 2.03 mm) and the VH (Bregma − 2.91 mm to − 3.39 mm) were selected based on the stereotaxic atlas of Paxinos and Franklin (4th edition). For light microscopy, sections were first quenched with 2% H_2_O_2_ in 70% methanol for 10 min, then incubated for 1 h in a blocking solution containing 10% fetal bovine serum, 3% bovine serum albumin, and 1% Triton X-100. Afterward, sections were incubated with an anti-IBA1 antibody (ionized calcium-binding adapter molecule 1, 1:1000 in blocking buffer, cat# 019-19741, FUJIFILM Wako Chemical) at 4 °C overnight. The following day, the antibody was washed out and the sections were incubated with biotinylated goat anti-rabbit polyclonal secondary antibody (1:300 in TBS cat# 111-066-046, Jackson ImmunoResearch) in Tris-buffered saline (TBS; 50 mM) for 1.5 h, followed by avidin–biotin complex solution (1:1000 in TBS; cat# PK-6100, Vector Laboratories) for 1 h at room temperature. Staining was revealed in Tris buffer (TB: 0.05 M, pH 8) containing 0.05% diaminobenzidine (DAB; cat# D5905-50TAB, Merck KGaA Darmstadt) and 0.015% hydrogen peroxide. Sections were mounted onto glass slides, dehydrated in ascending concentrations of ethanol, cleared in CitriSolV, and coverslipped with distyrene, plasticizer, and xylene (DPX) mounting medium (Electron Microscopy Sciences; EMS cat# 13510).

For SEM, sections were immunostained as mentioned above except that quenching was first done with 0.3% H_2_O_2_ in PBS for 5 min and then with 0.1% NaBH_4_ for 30 min, while the blocking buffer and antibody incubation solutions contained 0.01% Triton X-100. Following the immunostaining, sections post-fixed flat in osmium-thiocarbohydrazide-osmium. Briefly, sections were incubated in 3% ferrocyanide (cat# PFC232.250, BioShop) diluted in water combined (1:1) with 4% aqueous osmium tetroxide (cat#19170, Electron Microscopy Sciences) for 1 h, in 1% thiocarbohydrazide diluted in water (cat# 2231-57-4, Electron Microscopy Sciences) for 20 min, in 2% osmium tetroxide diluted in water for 30 min, then dehydrated in ascending concentration of ethanol followed by 3 propylene oxide washes of 5 min each. After post-fixation, brain sections were submerged overnight in Durcupan ACM resin (cat# 44611-44614, Merck KGaA Darmstadt). The following day, the brain sections were placed between 2 fluoropolymer sheets (ACLAR; cat# 50425-25, Electron Microscopy Sciences) covered with a thin layer of resin and placed for 72 h at 55 °C to polymerize. The regions of interest, CA1 SR, of the dorsal and ventral hippocampus were excised from the flat-embedded sections on ACLAR® sheets and glued to the top of resin blocks. Ultrathin sections (~ 75 nm) were generated with an ultramicrotome (Ultracut UC7 ultramicrotome, Leica Biosystems), collected on a silicon nitride chip, and glued on specimen mounts for SEM.

#### Density and morphology analysis

Light microscopy images were acquired in the DH (Bregma − 1.55 mm to − 2.03 mm) and the VH (Bregma − 2.91 mm to − 3.39 mm) CA1 SR of 6 mice using an Infinity 2 camera (5 MP; Lumenera), at 20× for the cellular density and spacing analysis, and at 40× for the morphology analysis. In the density analysis, the CA1 SR was first delineated using the freehand selection tool, based on the stereotaxic atlas of Paxinos and Franklin (4th edition), next, its area was measured in pixels and converted into mm^2^. Microglial density and distribution were determined using 10 to 12 sections/mice from 6 mice. The images were blindly analyzed using the ImageJ software (NIH, v.1.50b) as previously reported [55]. The ImageJ Analyze Particle function was used to count IBA1^+^ cells [56]. The density value of each region of interest (ROI) was determined as the total number of cells divided by the total area (cells/mm^2^). Distribution of IBA1^+^ cells was studied by measuring the average distance of each positive cell to its closest neighbor. Specifically, the nearest neighbor distance (NND) was obtained using the Nearest Neighbor Distance Plugin by Yuxiong Mao. The spacing index (arbitrary unit, a.u.) value was computed from the multiplication of the microglial density by the square average of NND per ROI and animal. For the microglial morphology analysis, 16 to 20 cells/animal in 6 mice were analyzed using a semi-automatic method adapted from previous research in our lab [57]. In each microglial cell, soma, and microglia arborization were manually traced using the freehand and polygon tools to obtain area values as well as shape descriptors, described as manual arbor. Next, an unsharp mask of each cell was obtained and manually corrected when needed. Area of the cell and shape descriptors (i.e., circularity and solidity) values were measured and identified as automated arbor, as previously described [57]. Briefly, (i) arbor circularity was calculated through 4*π* × (area/perimeter^2^), where a value of 1.0 represents a perfect circle and 0.0 an elongated shape; (ii) solidity was obtained by dividing the cell area by the convex cell area, with a value of 0.0 indicating a porous shape and 1.0 a convex shape; (iii) the aspect ratio was defined by dividing the major axis of the cell by the minor axis of the cell, where values higher than 1.0 equate to more elongated cell shapes; (iv) roundness was obtained by dividing the area of the arbor to the area of a circle with the same convex perimeter, with values closer to 1.0 representing more circular cell shapes. The mask of the cell was skeletonized and analyzed using a Skeleton 2D/3D Plugin to determine the number, average length, and maximal length of branches. The mask of each cell was converted into an outline to perform fractal analysis using the FracLac for ImageJ Plugin (https://imagej.nih.gov/ij/plugins/fraclac/FLHelp/Introduction.htm). Fractal dimension and lacunarity were extracted based on the cell’s contour, and a summary of the calculations is available in the reference guide (https://imagej.nih.gov/ij/plugins/fraclac/FLHelp/StartUpScreen.html). Briefly, the fractal dimension is an index for the complexity of the cell morphology, increasing in proportion to the repetition of a scale-invariant pattern, thus, the pixel detail [58]. Similarly, lacunarity refers to the gaps in the image, the more heterogeneous they are, the higher the lacunarity [58]. Fractal dimension and lacunarity are complementary, the first being particularly sensitive to morphology in whole cells, and the latter to features such as soma size and process length [59]. Higher values for lacunarity index and fractal dimension are indicative of more complex organization of branching and thus cellular morphologies associated with more ramified morphological states [55, 56]. The analysis was done with the absolute and relative values obtained per cell and averaged per animal as the final sample size.

#### Ultrastructural analysis

Electron microscopy imaging was performed in the DH (Bregma − 1.55 mm to − 2.03 mm) and the VH (Bregma − 2.91 mm to − 3.39 mm) CA1 SR of 4 mice, examining 8 to 12 microglial cell bodies in each animal imaged at 5 nm of resolution using a Crossbeam 540 field emission SEM with a Gemini column (Zeiss). The analysis was done blinded to the animal and hippocampal pole using QuPath, adapted for use in previous work from our lab [57]. Microglial cell bodies were identified based on their positive staining for IBA1 and their unique ultrastructure, notably presenting smaller cell bodies and nuclei than neighboring astrocytes or neurons, a characteristic heterochromatin pattern, as well as long stretches of endoplasmic reticulum (ER) [60]. Microglial contacts with other cell bodies (i.e., astrocytes, neurons, oligodendrocytes), as well as myelinated axons, blood vessels, and synaptic elements (pre-synaptic axon terminals and post-synaptic spines) were quantified [61]. Neurons were characterized by their pale nuclei and cytoplasm, frequent direct contacts with pre-synaptic terminals, whereas astrocytic cells were identified by their pale nuclei with a thin rim of heterochromatin and pale irregular cytoplasm [62]. Oligodendrocytes were recognized by their rectangular-shape cytoplasm, wider nuclear membrane spaces, short stretches of ER, and darker nuclei among other features [62]. Pre-synaptic axon terminals were identified by their synaptic vesicles, while post-synaptic spines were in contact with a pre-synaptic axon terminal and displayed a visible post-synaptic density [62]. Extracellular space pockets were characterized by clear spaces surrounding microglia, without delineating membranes [63], while extracellular digestion was recognized by extracellular space pockets containing debris in the vicinity of a microglial cell body [64]. Additionally, we characterized the health or functional state of microglial ER/Golgi apparatus, lysosomes, lipofuscin, mitochondria, and phagosomes in all the examined microglia [61]. Dilation of the ER/Golgi apparatus was identified when the distance between cisternal membranes was greater than 50 nm [65]. Mitochondria were considered elongated when their length was greater than 1 μm and they were altered when presenting vacuoles or changes in their cristae membranes [61]. Immature lysosomes, which comprise both primary and secondary lysosomes, were identified by their dense heterogeneous contents enclosed by a single membrane, sometimes associated with a phagosome [57]. Mature lysosomes were characterized by their larger size and contacts with lipofuscin (residual bodies or waste products resulting from previous phagocytosis), identified by their oval structure and finely granular composition [57]. Phagosomes were defined by their ovoid shape with a single membrane, clear interior and they were classified as empty or filled with contents [61]. Autophagosomes were further identified by the presence of digested elements inside doubled-membrane vacuoles with a clear interior [66].

The analysis was done with the absolute (e.g., number of cells with particular features) and relative (e.g., percentage of cells with particular features) values averaged per animal as the final sample size [57]. Of note, all microglial cells analyzed made direct contacts with pre-synaptic elements, resulting in a relative value of 100%, which was not included in the statistical analysis.

### Total RNA extraction from the whole DH and VH and RT real-time PCR

RNA from whole dorsal and ventral poles of the hippocampus of CTRL mice was isolated using GenElute™ Mammalian Total RNA Miniprep Kit and DNASE70-On-Column DNase I Digestion Set (Merck KGaA, Darmstadt, Germany) as previously described [67].

RNA quality and yield were verified using the NANODROP system (Thermo Fisher Scientific). Reverse transcription reaction was performed in a thermocycler (SimplyAMP, Thermo Fisher Scientific) using High Capacity cDNA Reverse Transcription Kit (Thermo Fisher Scientific) according to the manufacturer’s instructions under the following conditions: incubation at 25 °C for 10 min, reverse transcription at 37 °C for 2 h, inactivation at 85 °C for 5 min. Real time polymerase chain reaction (rt-PCR) was carried out on CFX Opus Real-Time PCR machine (Bio-Rad Laboratories), using SsoAdvanced Universal SYBR Green Supermix (Bio-Rad Laboratories). Specific forward and reverse primers, designed and verified with the primer-BLAST designing tool, were used at a final concentration of 300 nM (Additional file 1: Table S1 for primer sequences). The PCR protocol consisted of 95 °C for 30 s; 40 cycles of 95 °C for 15 s, 60 °C for 30 s. PCR products were subjected to a melting curve analysis to verify the absence of artifacts or non-specific products. Cycle threshold (Ct) value was determined by the CFX maestro software (Bio-Rad Laboratories). Gene expression was calculated with the comparative cycle threshold (ΔΔCt) method using Cyclophilin A (*CypA*) as an endogenous control and the results were reported as fold change compared to average values of the dorsal portion of the hippocampus.

### Isolation of CD11b^+^ and CD11b^−^ cells from the hippocampus and RT real-time PCR

CTRL mice were anesthetized by inhalation of halothane (Merck KGaA, Darmstadt, Germany), perfused with saline to eliminate circulating peripheral immune cells and decapitated. DH and VH were isolated and cut into small pieces and single-cell suspension was achieved in Hank’s balanced salt solution (HBSS). The tissue was further mechanically dissociated using a glass wide-tipped pipette and processed for cluster of differentiation (CD)11b^+^ cells extraction with a magnetic-activated cell sorting (MACS) system [68]. Briefly, CD11b^+^ cells were magnetically labeled with CD11b MicroBeads. The cell suspension was loaded onto a MACS Column placed in the magnetic field of a MACS Separator and the negative fraction was collected. After removing the magnetic field, CD11b^+^ cells were eluted as the positive fraction. Live CD11b^+^ cells were assessed by flow cytometry and the purity was ~ 99% [68].

After sorting the positive and the negative fractions, RNA was isolated with the RNeasy Mini Kit and processed for rt-PCR (Qiagen). The quality and yield of RNAs were verified using the NANODROP One system (Thermo Fisher Scientific). Reverse transcription reaction of CD11b^+^ and CD11b^−^ cells collected by MACS was performed in a thermocycler (MJ Mini Personal Thermal Cycler; Bio-Rad Laboratories) using IScript TM Reverse Transcription Supermix (Bio-Rad Laboratories) according to the manufacturer’s protocol, under the following conditions: incubation at 25 °C for 5 min, reverse transcription at 42 °C for 30 min, inactivation at 85 °C for 5 min. rt-PCR was carried out in a I-Cycler IQ Multicolor rt-PCR Detection System (Bio-Rad Laboratories) using SsoFast EvaGreen Supermix (Bio-Rad Laboratories) according to the manufacturer’s instructions. The PCR protocol consisted of 40 cycles of denaturation at 95 °C for 30 s and annealing/extension at 60 °C for 30 s. For quantitative analysis, the comparative threshold cycle (*C*_t_) method was used. The *C*_t_ values from each gene were normalized to the *C*_t_ value of glyceraldehyde 3-phosphate dehydrogenase (*Gapdh*) in the same RNA samples. Relative quantification was performed using the 2^−ΔΔCt^ method and the results were reported as fold change compared to average values of the dorsal portion of the hippocampus expressed as fold change in arbitrary values. The primers used are listed in Additional file 1: Table S1. For consistency check, part of the experiments on sorted cells was repeated in different cohorts of CTRL animals obtained from the laboratories of the University of Modena and Reggio Emilia and Rome, blindly analyzed, and then pooled. Raw CT values for the CD11b^+^ and CD11b^−^ cell fraction and whole VH and DH are reported in Additional file 1: Table S2.

### Statistical analysis

Electrophysiological and gene expression data were analyzed using SigmaPlot software, statistical significances were assessed by Student’s *t*-test, one-way ANOVA (for electrophysiology), and paired Student’s *t*-test (for gene expression analysis). Normality was verified with Shapiro–Wilk test and Mann–Whitney test was utilized for non-normally distributed data sets. Post hoc comparisons were performed with Holm–Sidak method. For statistical analysis of potassium currents expressed by microglia cells, statistical difference of proportions was obtained with *z*-test. Two-way ANOVA repeated measure was used to assess the significance between the current–voltage relationship and current densities. All data are expressed as mean ± standard error of mean (S.E.M.), *N*/*n* refers to the number of slices on the total number of mice analyzed.

Density and spacing data were analyzed using the GraphPad Prism software (v.9). A Shapiro–Wilk normality test was applied to evaluate normality of data distribution. Paired parametric Student’s *t*-tests were performed to analyze the data, which was all normally distributed. Morphology and ultrastructure data were analyzed using GraphPad Prism (v.7). Normality was verified with Shapiro–Wilk test. Comparisons of morphology and ultrastructure were done using paired two-tailed parametric Student’s *t*-test for normally distributed data sets or nonparametric Wilcoxon tests for non-normally distributed data sets. All data are expressed as mean ± S.E.M., *n* refers to the number of animals analyzed.

Levels of significance were set to: *p < 0.05, **p < 0.01, ***p < 0.001.

## Results

### Long-term plasticity is differentially modulated by microglia in the DH compared to the VH

We have previously demonstrated that plasticity differs, in mice, between hippocampal poles, being higher in the DH compared to the VH [21].

**Fig. 1:**
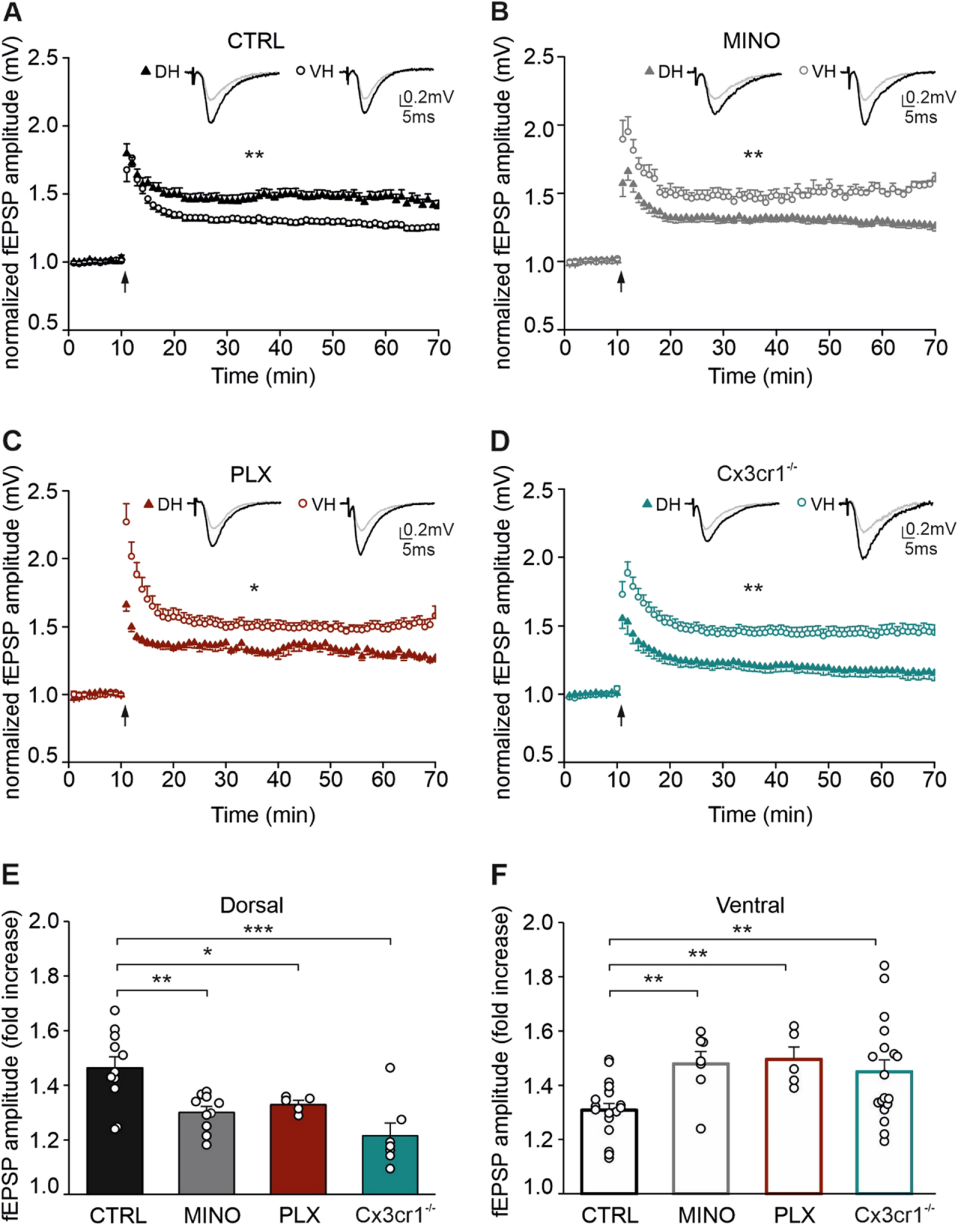
Hippocampal LTP in the two poles is influenced by microglia. Time course of normalized averaged amplitudes of fEPSPs recorded from CA1 region of dorsal and ventral hippocampal slices in **A** control, **B** minocycline, **C** PLX5622, **D** *Cx3cr1*^−/−^ groups following Schaffer collateral high-frequency stimulation (HFS, 2 trains of stimuli at 100 Hz of 1 s duration each, 3 s inter-train interval, arrow). Symbols represent mean ± S.E.M. of normalized fEPSP amplitude evoked every 20 s. Above are representative traces taken before (grey) and after (black) HFS from each region. Comparison of the mean LTP amplitude, recorded at 35 min after HFS, between experimental groups in dorsal (**E**) and ventral (**F**) hippocampus. Dots represent individual slices. Statistical significance was assessed by *t*-test and one-way ANOVA, Holm–Sidak for post hoc comparison. *p < 0.05, **p < 0.01, ***p < 0.001

To investigate the possible roles of microglia in modulating short- and long-term synaptic plasticity of excitatory synapses made by Shaffer collaterals onto CA1 neurons in the DH and VH, we performed electrophysiological recordings on acute hippocampal slices while interfering with microglial functions. In detail, we measured changes in fEPSP amplitude with time following an HFS. As previously reported [21], we confirmed that in CTRL animals, the LTP amplitude measured 35 min after HFS was higher in the DH (1.463 ± 0.041, *N*/*n* = 13/12) compared to the VH (1.308 ± 0.024, *N*/*n* = 19/16; *p* = 0.002, *t* = 3.409; Fig. 1A). By contrast, in the slices treated in vitro with MINO to modulate microglial activity, we observed that LTP was higher in the VH (1.479 ± 0.045, *N*/*n* = 8/8) than in the DH (1.301 ± 0.021, *N*/*n* = 10/10; *p* = 0.001, *t* = − 3.886; Fig. 1B). Similarly, in mice treated with PLX to deplete microglia, the LTP amplitude was significantly higher in the VH (1.509 ± 0.043, *N*/*n* = 5/3) compared to the DH (1.318 ± 0.031, *N*/*n* = 5/3; *p* = 0.022, *t* = − 3.049; Fig. 1C). These results imply that modifying microglial functions differentially affects plasticity at the two hippocampal poles, decreasing LTP amplitude in the DH and increasing it in the VH.

In the brain, *Cx3cr1* is mostly expressed by microglia [69–71] while its ligand, *Cx3cl1*, is abundantly found on neurons. As the CX3CL1–CX3CR1 pathway modulates synaptic plasticity and mediates communication between neurons and microglia [37, 38, 69, 72–74], we explored whether the microglia-mediated effects on LTP described above involve this signaling pathway. Thus, we repeated the electrophysiological recordings on hippocampal slices from mice with the genetic deletion of the *Cx3cr1* gene. We observed that in the Cx3cr1^−/−^ mice, the amplitude of LTP was higher in the VH (1.450 ± 0.043, *N*/*n* = 21/12) compared to the DH (1.215 ± 0.046, *N*/*n* = 9/7; *p* = 0.005, *t* = − 3.075; Fig. 1D), resembling the results obtained with MINO and PLX treatments. These findings demonstrate that a perturbation of neuron–microglia communication impacts LTP amplitude in both hippocampal regions.

Furthermore, in the DH, comparison of LTP amplitude revealed a significant reduction by the different microglial modulation strategies compared to the control group (one-way ANOVA *p* = 0.005; Holm–Sidak: CTRL vs MINO *p* = 0.004, *t* = 3.419; CTRL vs PLX *p* = 0.037, *t* = 2.021; CTRL vs Cx3cr1^−/−^ *p* < 0.001, *t* = 4.739; Fig. 1E). By contrast, in the VH, all the experimental approaches significantly increased LTP amplitude compared to the control group (one-way ANOVA *p* ≤ 0.001; Holm–Sidak: CTRL vs MINO *p* = 0.019, *t* = 2.707; CTRL vs PLX *p* = 0.010, *t* = 2.694; CTRL vs Cx3cr1^−/−^ *p* = 0.012, *t* = 3.024; Fig. 1F).

**Fig. 2:**
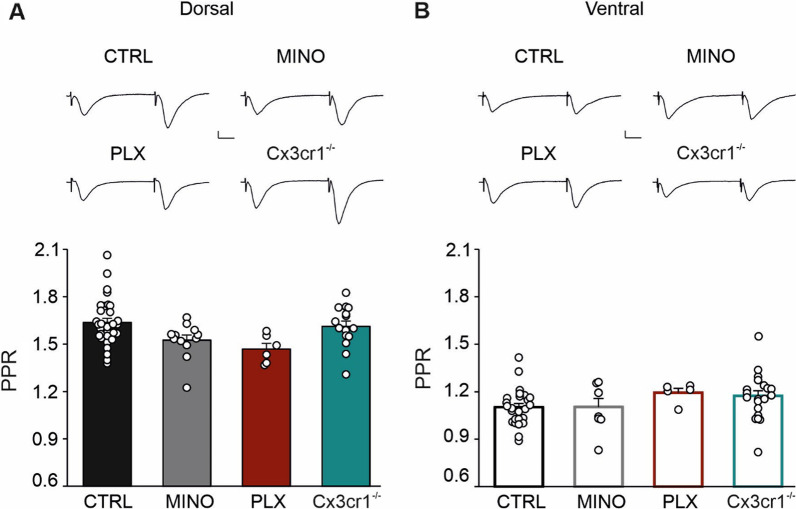
Paired-pulse ratio (PPR) is not affected by microglia. Bar histograms represent averaged PPR values for dorsal (**A**) and ventral (**B**) hippocampus in all experimental conditions. Above, fEPSP representative traces of PPR from DH and VH in all experimental conditions, scale bars: 0.2 mV, 10 ms. Data are shown as mean ± S.E.M., dots represent values from single slice. Note that PPR in the VH is reduced compared to the DH for all experimental conditions (***p < 0.001 not shown). Statistical significance was assessed using one-way ANOVA, Holm–Sidak for post hoc comparison

We then investigated the role of microglia in modulating short-term plasticity by measuring changes in the PPR, a form of short-term plasticity generally associated with changes in neurotransmitter release probability. In the CTRL group, we found that the PPR is increased in slices obtained from the DH (1.636 ± 0.027, *N*/*n* = 31/19) compared to the VH (1.102 ± 0.023, *N*/*n* = 25/18; *p* < 0.001; Fig. 2A, B), confirming previous findings [21]. Similarly, in all experimental conditions, PPR in the DH was higher compared to the VH (*p* < 0.001; Fig. 2A, B), while treatment with MINO (DH 1.524 ± 0.032, *N*/*n* = 12/10; VH 1.104 ± 0.052, *N*/*n* = 8/8) or with PLX (DH 1.468 ± 0.036, *N*/*n* = 6/3; VH 1.194 ± 0.027, *N*/*n* = 5/3), and *Cx3cr1* knocking out (DH 1.612 ± 0.034, *N*/*n* = 15/10; VH 1.174 ± 0.031, *N*/*n* = 21/12) showed comparable PPR with respect to the CTRL at the two poles. Since PPR depends on a transient increase of transmitter release due to residual pre-synaptic calcium, these data suggest that under basal conditions microglia do not participate in regulating the differences in release probability observed at Schaffer collateral–CA1 synapses between the VH and the DH.

Taken together, these findings indicate that microglia act in a region-specific manner to regulate hippocampal LTP, and that the CX3CL1–CX3CR1 pathway plays a key role in LTP modulation.

**Fig. 3:**
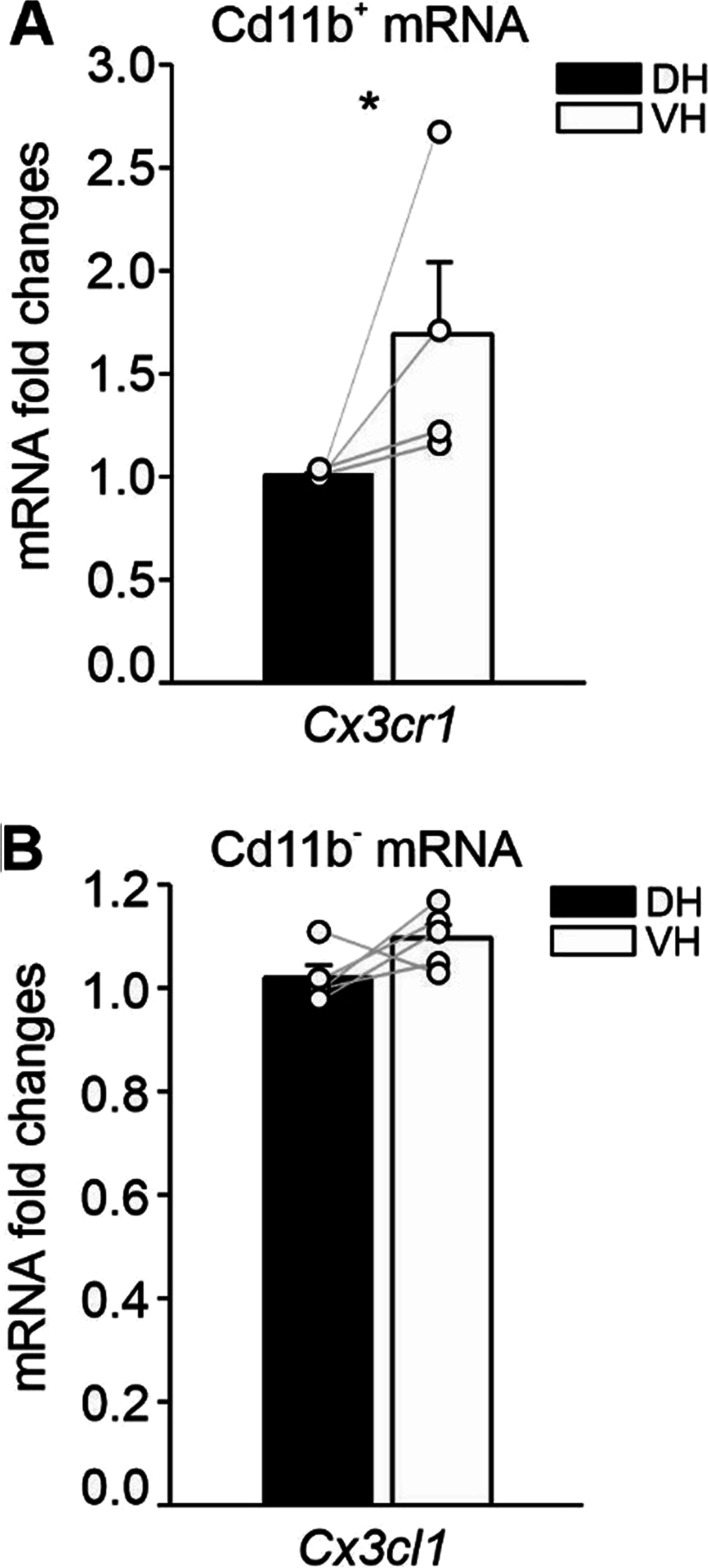
CX3CR1 is overexpressed in CD11b^+^ cells from VH versus DH. **A** RT-PCR of *Cx3cr1* gene expression on CD11b^+^ cells sorted from DH and VH of CTRL mice. **B** RT-PCR of *Cx3cl1* gene expression on CD11b^−^ cells sorted from DH and VH of CTRL mice. Data are shown as mean ± S.E.M. with dots representing values from single mice. Statistical significance was assessed using paired *t*-test. *p < 0.05

We have previously demonstrated that in VH the absence of *Cx3cr1* increases LTP, whereas treatment with CX3CL1 inhibits it [37, 38, 75]. Here, we investigated whether the effects described above could be due to a differential *Cx3cr1* expression at the two poles. We found that *Cx3cr1* mRNA levels were higher in CD11b^+^ cells isolated from the VH compared to the DH (DH 1.00 ± 0.010, VH 1.680 ± 0.349, *n* = 4; *p* = 0.029; Fig. 3A) while the expression level of the ligand (*Cx3cl1*) in CD11b^−^ cells did not differ between the hippocampal poles (DH 1.022 ± 0.022, VH 1.098 ± 0.025, *n* = 5; Fig. 3B), supporting an inverse relationship between LTP levels and the expression of *Cx3cr1* in CTRL animals.

### The expression of plasticity-related target genes differs between the hippocampal poles

In the brain, cytokines and neurotrophins play an important role in modulating LTP, plasticity, and memory processes under physiological conditions. A cytokine network that includes IL-1β, IL-6, TNF-α and neurotrophins, such as brain-derived neurotrophic factor (BDNF), co-operates to modulate hippocampal LTP expression under normal physiological conditions [36].

We thus measured the expression levels of *Il-1β*, *Il-6*, *Tnf-α*, and *Bdnf* in total RNA extracts from whole DH and VH, as well as in CD11b^+^ or CD11b^−^ cells isolated from the DH and VH of CTRL animals.

**Fig. 4:**
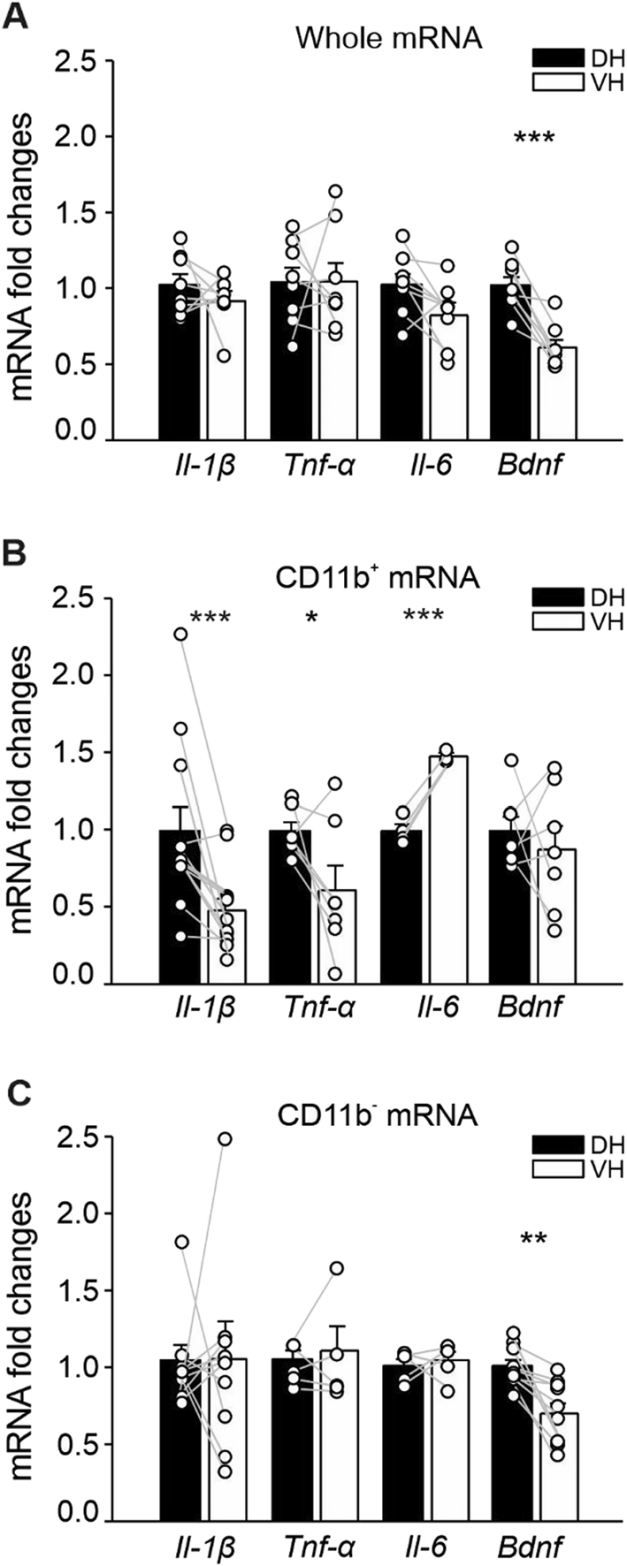
Molecules involved in plasticity are differentially expressed in the two hippocampal poles. Gene expression of *Il-1β, Tnf-α, Il-6* and *Bdnf* in total mRNA extracted from the whole dorsal and ventral hippocampus (**A**), in CD11b^+^ cells (**B**), and CD11b^−^ cells (**C**) sorted from DH and VH of CTRL mice. Data are shown as mean ± S.E.M., dots represent values from single mice. Statistical significance was assessed by paired *t*-test. *p < 0.05, **p < 0.01, ***p ≤ 0.001

Expression levels of *Il-1β*, *Tnf-α* and *Il-6* did not differ between the VH and the DH when considering their whole-cell populations (*Il-1β*: DH 1.017 ± 0.073, VH 0.909 ± 0.065, *n* = 8; *Tnf-α*: DH 1.034 ± 0.096, VH 1.039 ± 0.120, *n* = 8; *Il-6*: DH 1.018 ± 0.071, VH 0.815 ± 0.084, *n* = 8; Fig. 4A). When we analyzed the expression of these same genes in isolated CD11b^+^, we found that *Il-1β* and *Tnf-α* mRNAs were significantly lower in the VH compared to the DH (*Il-1β*: DH 1.000 ± 0.157, VH 0.478 ± 0.078, *n* = 12; *p* = 0.001, *Tnf-α*: DH 1.000 ± 0.056, VH 0.609 ± 0.163, *n* = 7; *p* = 0.043, *t* = 2.267; Fig. 4B) while *Il-6* expression was higher in the VH compared to the DH (DH 1.001 ± 0.042, VH 1.488 ± 0.221, *n* = 4; *p* < 0.001, *t* = − 12.169; Fig. 4B). By contrast, *Bdnf* transcript levels were lower in the VH when considering all the cell populations (DH 1.011 ± 0.056, VH 0.604 ± 0.049, *n* = 8; *p* < 0.001, *t* = 5.584; Fig. 4A) but not in CD11b^+^ cells (DH 1.000 ± 0.093, VH 0.878 ± 0.156, *n* = 7; Fig. 4B). Measuring the expression levels of these cytokines within the CD11b^−^ fraction we did not reveal any *Il-1β*, *Tnf-α* and *Il-6* expression differences between the two poles (*Il-1β*: DH 1.038 ± 0.098, VH 1.043 ± 0.244, *n* = 10; *Tnf-α*: DH 1.042 ± 0.059, VH 1.100 ± 0.156, *n* = 5; *Il-6*: DH 1.000 ± 0.045, VH 1.036 ± 0.055, *n* = 5; Fig. 4C) while *Bdnf* expression was increased in the DH versus VH (DH 1.000 ± 0.0392, VH 0.693 ± 0.0642, *n* = 10, *p* = 0.002, *t* = 4.214; Fig. 4C).

These results suggest that different levels of microglia-released cytokines, such as IL-1β, TNF-α, IL-6, as well as BDNF of neuronal or astrocytic origin, could contribute to setting up the distinct LTP amplitude in the DH and VH under normal physiological conditions.

### Microglia density and distribution significantly differ between the DH and the VH

**Fig. 5:**
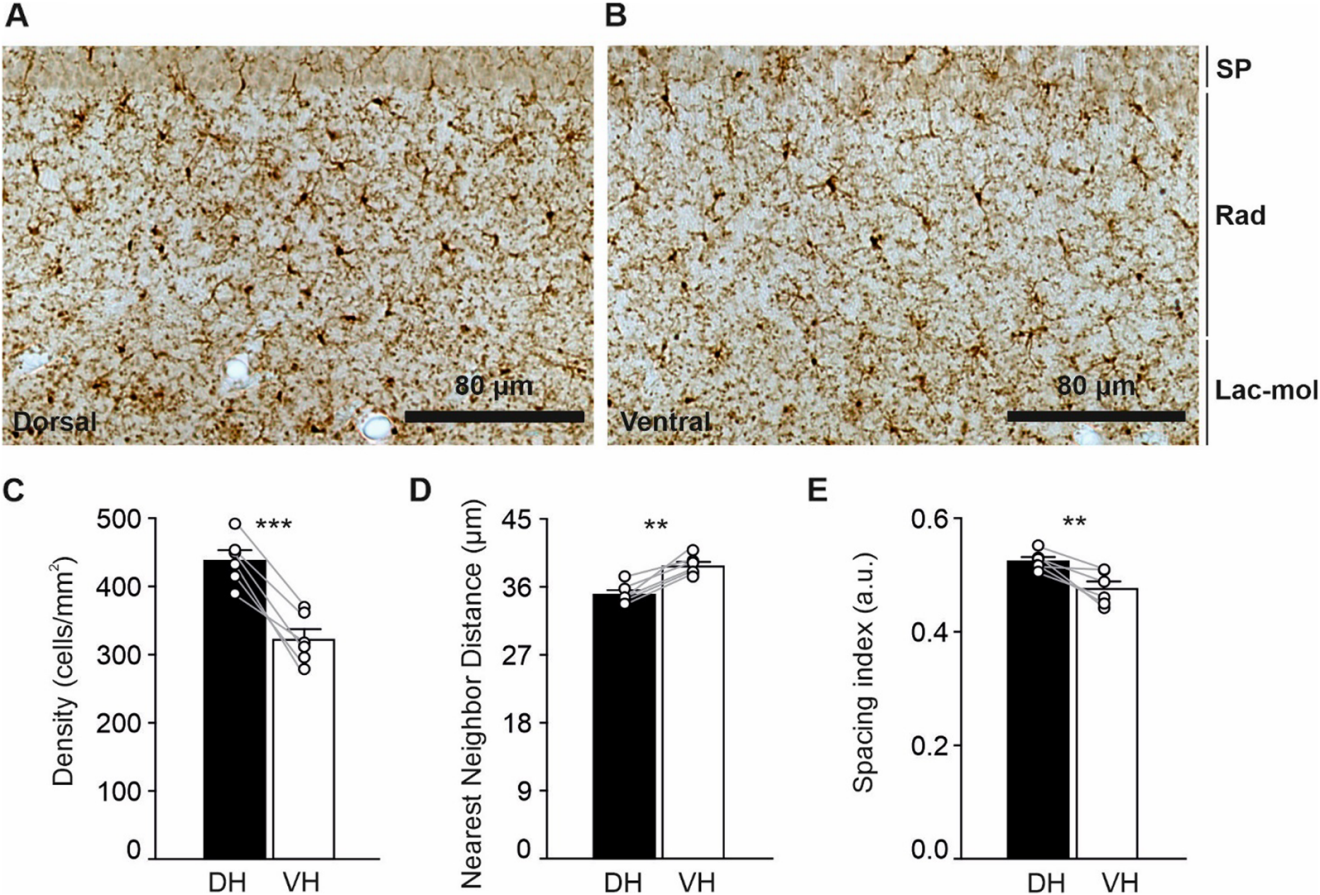
Variations in microglial density and distribution in the DH versus VH CA1 *stratum radiatum*. Representative brightfield images at a ×20 magnification illustrating IBA1^+^ microglia in the DH (**A**) and VH (**B**) CA1 SR. The DH had a higher microglial density compared to the VH (**C**). Accordingly, the NND was decreased (**D**) and the spacing index also differed between the two hippocampal poles (**E**). Data are expressed as mean ± S.E.M. Statistical significance was assessed using a paired Student’s *t*-test (*n* = 6 mice). *p-value < 0.1, **p-value < 0.01, and ***p-value < 0.001. a.u.: arbitrary units; DH: dorsal hippocampus; Lac-mol: *stratum lacunosum-moleculare*; NND: nearest neighbor distance; Rad: *stratum radiatum*; S.E.M.: standard error of the mean; SP: pyramidal cell layer; VH: ventral hippocampus

To investigate possible regional differences in the density and distribution of microglia, we performed an immunoperoxidase staining for IBA1 to reveal microglia/macrophage cells [56, 76] and quantified all the positive cells in the CA1 *stratum radiatum* (SR) (Fig. 5A, B). We found a significantly higher density of IBA1^+^ cells in the DH compared to the VH (DH 436.8 ± 14.29 cells/mm^2^; VH 321.2 ± 14.69 cells/mm^2^, *n* = 6, *p* = 0.0003, *t* = 9.10; Fig. 5C and Additional file 1: Table S3). In agreement with an increased density of IBA1^+^ cells, the analysis of nearest neighbor distance (NND) showed a reduction in the DH versus VH (DH 34.76 ± 0.537, VH 38.48 ± 0.526 µm, *n* = 6, *p* = 0.0011, *t* = 6.75; Fig. 5D) with a spacing index significantly different in the DH compared to the VH (DH 0.521 ± 0.006 a.u.; VH 0.472 ± 0.012 a.u., *n* = 6, *p* = 0.0066, *t* = 4.45; Fig. 5E).

These results show differences in the number and distribution of IBA1^+^ cells between the two hippocampal poles under normal physiological conditions.

### Microglial morphology and ultrastructure significantly differ between the DH and the VH

**Fig. 6:**
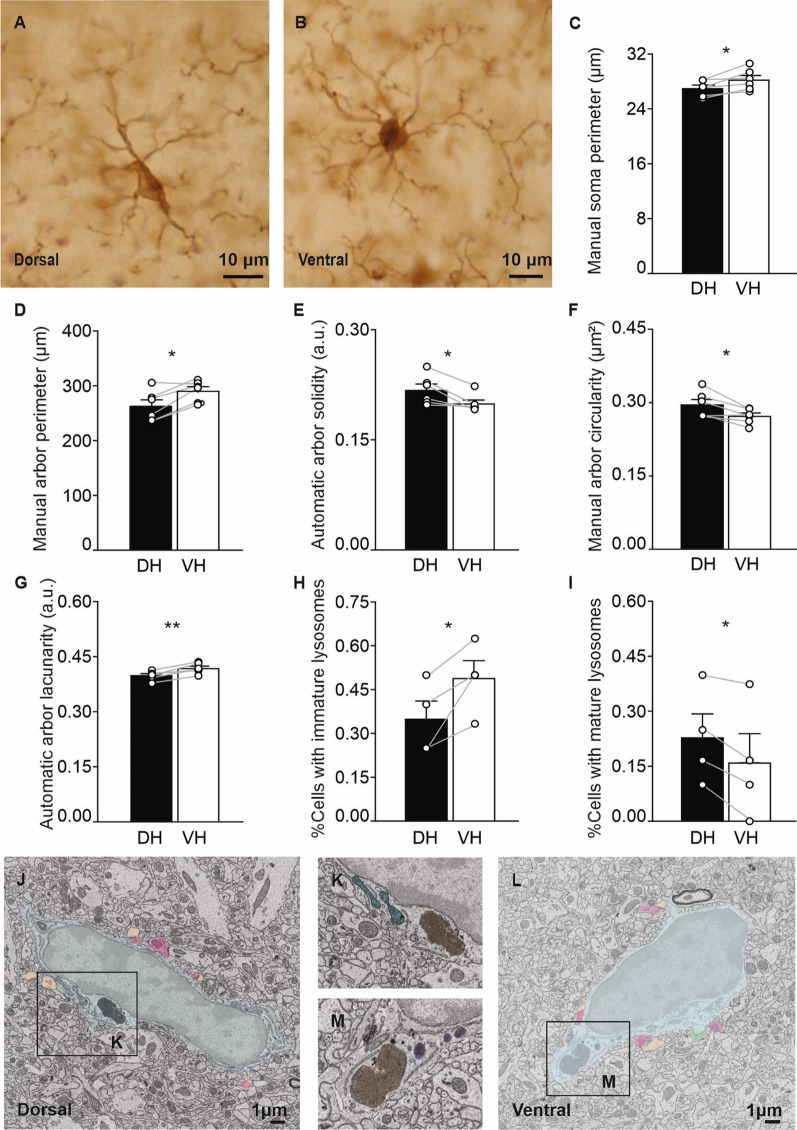
Variations in microglial morphology and ultrastructure in the DH versus VH CA1 *stratum radiatum*. Representative brightfield images at a ×40 magnification showing IBA1^+^ microglia in the dorsal (**A**) and ventral (**B**) CA1 *stratum radiatum* (SR). The soma (**C**) and manual arbor perimeters (**D**), as well as the automatic arbor solidity (**E**), manual arbor circularity (**F**) and lacunarity (**G**), were calculated for IBA1^+^ cells in the DH and VH using brightfield microscopy (*n* = 6 mice). The mean relative number of microglial cell bodies containing immature (**H**) and mature (**I**) lysosomes per animal were determined using scanning electron microscopy (SEM) (*n* = 4 mice). Representative SEM of microglia in the dorsal (**J**, **K**) and ventral (**L**, **M**) CA1 SR. SEM images are pseudocolored as follows: light blue, microglia; grey, microglial nucleus; green, extracellular digestions; beige, dendritic spine; pink, axon terminal; dark blue, altered mitochondria; purple, immature lysosomes; and yellow, mature lysosomes. Data are expressed as mean ± S.E.M. For normally distributed data, two-tailed paired Student’s *t*-test was used (**C**, **D**, **F**–**I**), while for non-normally distributed data the paired Wilcoxon test was used (**E**). Levels of significance were set to: *p < 0.05, **p < 0.01

Microglial morphology is tightly linked to their function [74, 75]. To assess possible differences in microglial function between the DH and VH, we next performed morphology (Fig. 6A, B, Additional file 1: Table S4) and ultrastructure analyses of IBA1^+^ cells in CTRL mice (Fig. 6J–M, Additional file 1: Table S5). For morphology analysis, a semi-automated approach was used. The cell body area and perimeter were manually traced and measured, as well as a variety of shape and arborization descriptors complementarily obtained via a manual arbor and an automated arbor mask in IBA1^+^ cells comparing the CA1 SR of the DH and the VH. A significantly increased soma perimeter (DH 27.050 ± 0.470, VH 28.298 ± 0.631 µm, *n* = 6, *p* = 0.023, *t* = 3.222; Fig. 6C) and manual arbor perimeter (DH 263.482 ± 11.433, VH 291.202 ± 7.796 µm; *n* = 6, *p* = 0.01, *t* = 4.02; Fig. 6D) were found, accompanied by a reduction in automatic arbor solidity (DH 0.218 ± 0.008, VH 0.195 ± 0.004; *n* = 6, *p* = 0.031; Fig. 6E) and manual arbor circularity (DH 0.295 ± 0.010, VH 0.271 ± 0.006 a.u., *n* = 6, *p* = 0.024, *t* = 3.189; Fig. 6F), in IBA1^+^ cells of the VH versus DH. Increased perimeters are indicative of more cell ramification, enhancing the convex hull area of the cell and decreasing its density or solidity [77], as well as circularity, as observed in the VH. Moreover, to investigate cell morphology complexity and branching, the lacunarity and fractal dimension of IBA1^+^ cells were measured. The lacunarity index of IBA1^+^ cells was significantly higher in the VH versus DH (DH 0.398 ± 0.004, VH 0.418 ± 0.005 a.u.; *n* = 6, *p* = 0.002, *t* = 5.503, Fig. 6G). Lacunarity is used to infer gaps in a specific shape, therefore, the more heterogeneous or rotationally variant a cellular shape is, the higher the lacunarity [55]. The observed increase in lacunarity thus reveals that microglia are more ramified in the VH compared to the DH. In complement to light imaging and cell morphology analysis, SEM was used to perform in CTRL mice an ultrastructural analysis of microglia, which includes the classification and quantification of contacts with the parenchyma, as well as intracellular features in the CA1 SR of the DH and the VH (Fig. 6I–L). We found that microglia in the VH presented a higher prevalence of immature lysosomes comprising primary and secondary lysosomes associated with active phagocytosis (DH 0.350 ± 0.061, VH 0.489 ± 0.059; *n* = 4, *p* = 0.034, *t* = 3.694; Fig. 6H), whereas microglia in the DH had a higher abundance of mature lysosomes (DH 0.229 ± 0.064, VH 0.160 ± 0.079; *n* = 4, *p* = 0.023, *t* = 4.272; Fig. 6I), which presented lipofuscin inclusions (tertiary lysosomes or residual bodies resulting from previous phagocytosis), suggesting an increased phagocytic intake and activity in the VH.

However, we did not observe differences in filled or empty phagosomes between the DH and VH, indicating no difference in digestive ability (see Additional file 1: Table S5). For all the other parameters analyzed we did not observe changes between the VH and DH, including distribution of regular and dilated ER/Golgi apparatus cisternae, mitochondrial structure, autophagosomes, as well as microglial contacts with the neuropil, e.g., neuronal cell bodies, myelinated axons, pre-synaptic axon terminals, post-synaptic dendritic spines, astrocytes, blood vessels and extracellular space pockets (Additional file 1: Table S5).

**Fig. 7:**
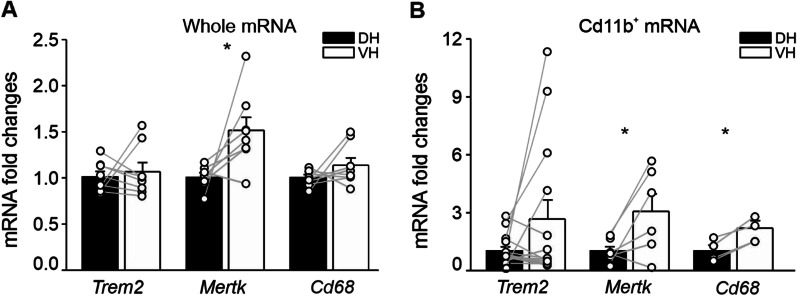
Phagocytic markers expression is increased in VH compared to DH. Gene expression of the phagocytic marker *Trem2*, *Mertk* and *Cd68* in total mRNA extracted from dorsal and ventral hippocampus (**A**), and in CD11b^+^ cells (**B**) sorted from the DH and the VH of CTRL mice. Data are shown as mean ± S.E.M., dots represent values from single mice. Statistical significance was assessed using paired *t*-test. *p < 0.05

We then analyzed the expression of phagocytic markers in both isolated microglial cells (CD11b^+^) and unsorted cell populations from the DH and the VH poles of CTRL mice. In unsorted cell populations, *Mertk* expression was higher in the VH compared to the DH (*Mertk*: DH 1.007 ± 0.049, VH 1.518 ± 0.143, *n* = 7; *p* = 0.047, *t* = − 2.494; Fig. 7A), whereas *Trem2* and *Cd68* expression did not differ between hippocampal poles (*Trem2*: DH 1.011 ± 0.059, VH 1.068 ± 0.099, *n* = 8; *Cd68* 1.003 ± 0.034, *n* = 7; VH 1.139 ± 0.078, *n* = 8; Fig. 7A). In CD11b^+^ cells, mRNA levels of *Mertk* and *Cd68* were higher in the VH compared to the DH (*Mertk*: DH 1.000 ± 0.207, VH 3.04 ± 0.900, *n* = 6; *p* = 0.050, *t* = − 2.548; *Cd68*: DH 1.000 ± 0.282, VH 2.178 ± 0.378, *n* = 4; *p* = 0.050, *t* = − 4.028; Fig. 7B), while *Trem2* showed only a tendency to be enhanced in the VH (DH 1.000 ± 0.197; VH 2.644 ± 0.983, *n* = 15; *p* = 0.119 Fig. 7B). Overall, these data indicate an increased expression of microglial phagocytic markers in the VH versus DH.

### Microglia of the ventral pole present increased outward rectifier K^+^ currents

Ions channels are involved in the regulation of many microglial functions [78], including shape changes migration and cytokine release. In particular, upregulation of inward rectifier K^+^ currents, outward K^+^ currents and calcium-activated K^+^ channels has been reported in activated microglia [79, 80]. In addition, K_v_1.3 channels has been reported to control microglial release of cytokines [81], although at present, the role of microglial K^+^ currents in physiological condition and its regional differences are still poorly understood.

**Fig. 8:**
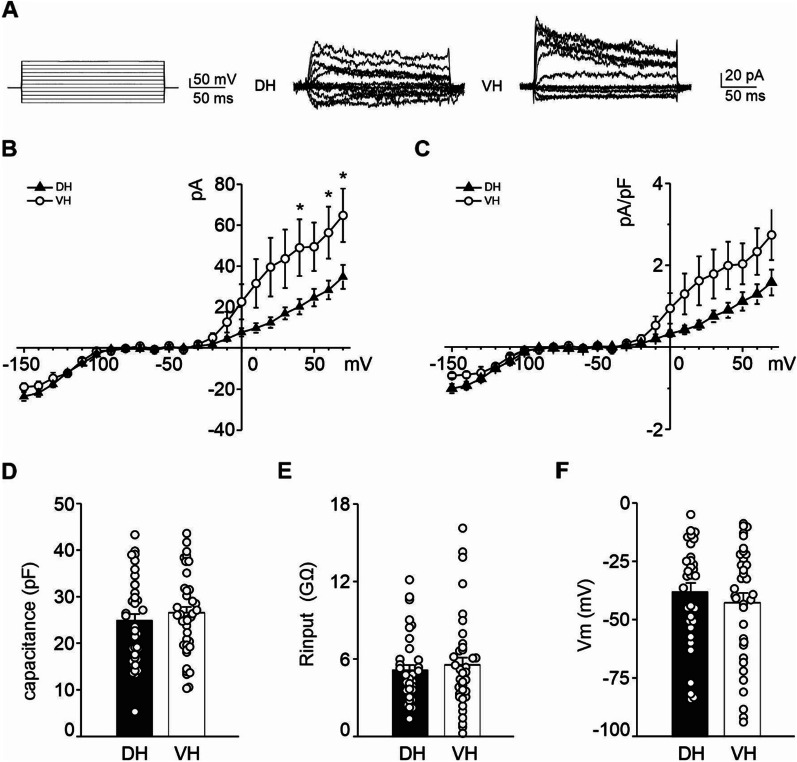
Outward K^+^ currents are enhanced in VH. **A** Representative steps and recordings from DH and VH microglia (odd sweeps were selected for graphical reasons). **B** Current–voltage relationship and **C** current densities–voltage relationship evoked in GFP^+^ microglia cells by voltage steps from − 150 to + 70 mV starting from the holding potential of − 70 mV. Mean histograms (± S.E.M.), with dots representing values from single cell of capacitance (**D**), input resistance (**E**), and resting membrane potential (**F**) measured from DH and VH microglia (*n* = 38 and 40, respectively). Statistical significance was assessed by two-way ANOVA RM. *p < 0.05

We analyzed, by whole-cell patch-clamp recordings, the electrophysiological properties of microglia from the dorsal and ventral CA1 hippocampal regions of Cx3cr1^+/GFP^ mice. We evaluated the expression of K^+^ currents in GFP^+^ microglial cells by measuring the currents evoked by voltage steps. We found that the current–voltage curves from the VH and the DH microglia diverge at positive potentials, with the peak of outward rectifier K^+^ currents being higher in the VH than the DH (*n* = 40 and 38, respectively, 40 mV *p* = 0.042; 60 mV *p* = 0.047; 70 mV *p* = 0.034, Fig. 8A, B). A similar trend was observed for current density measurement (pA/pF) although, in this case, post hoc comparison did not reveal any statistical differences (+ 70 mV *p* = 0.06; Fig. 8C). However, the proportion of microglia expressing outward K^+^ currents was similar between the two groups [VH 0.425 (17/40), DH 0.395 (15/38) *p* = 0.787, *Z* test]. The inward-rectifier K^+^ currents evoked by hyperpolarizing pulses from − 70 to − 150 mV did not differ between VH and DH microglia (− 18.26 ± 2.17 pA and 23.51 ± 2.14 pA, respectively). The passive properties of microglia appeared comparable between the two poles, with the capacitance values (DH 24.735 ± 1.522 GΩ; VH 26.400 ± 1.408 GΩ, Fig. 8D), the input resistance (DH 5.109 ± 0.418 pF; VH 5.541 ± 0.566 pF, Fig. 8E), and the resting membrane potential (DH − 38.247 ± 3.919 mV; VH − 42.785 ± 4.209 mV, Fig. 8F) being similar in VH compared to DH microglia. The different expression levels of outward rectifier K^+^ channels suggest a distinct basal phenotypic state of microglia in the CA1 SR region across the two hippocampal poles.

## Discussion

The hippocampus strongly differs along the longitudinal axis at structural, molecular, as well as functional levels [2, 9, 15, 20, 22, 82]. We previously demonstrated that LTP is higher in the dorsal part of the hippocampus and declines towards the ventral part [21]. Since microglia play an important role in maintaining brain homeostasis by regulating the structural and functional properties of neuronal networks, including synaptic plasticity [42], we investigated whether, under physiological conditions, the difference in hippocampal VH and DH plasticity could rely on differences in microglial properties. We demonstrate that microglia show unique morphological, ultrastructural, and physiological features between the two hippocampal poles and that the depletion of microglia by PLX, their modulation by MINO, or the absence of the *Cx3cr1* gene, strongly affect LTP in a region-specific manner, indicating that the proper chemokine signaling between neurons and microglia is crucial for the maintenance of physiological plasticity mechanisms along the longitudinal axis of the hippocampus.

### Role of CX3CL1–CX3CR1 axis and plasticity markers in setting LTP levels at the two poles

Recent evidence demonstrates that hippocampal synaptic strength and plasticity changes can arise from signaling in immune-related pathways, including those modulated by microglia. Among these, the interaction of the microglial fractalkine receptor, CX3CR1, with its ligand, the neuronal CX3CL1, allows for precise communication between neurons and microglia, as well as variations in neuronal activity [72, 73]. The disruption of CX3CL1–CX3CR1 signaling indeed underlies alteration of circuits development, synaptic maturation, cognitive function, plasticity processes, responses to the environment, and pathogenesis of central nervous system disorders [37, 38, 41, 51, 54, 71, 74, 83]. Moreover, we have previously shown that neuronal CX3CL1 inhibits LTP through adenosine receptor type 3 in wild-type mice but not in *Cx3cr1*^*−/−*^ mice [75] and that mice lacking CX3CL1–CX3CR1 signaling show enhanced LTP compared to wild-type mice [37, 38], indicating that this signaling pathway is important for setting the level of hippocampal LTP.

In this study we further investigated the role of CX3CL1–CX3CR1 axis in modulating synaptic plasticity specifically in the DH and VH. We first confirmed that in basal conditions LTP is higher in the DH compared to VH. Interestingly, in *Cx3cr1*^*−/−*^ mice, we observed an opposite behavior, with LTP being increased in VH and reduced in DH with respect to CTRL mice. The same outcome was observed when microglia were believably modulated by MINO, a tetracycline derivative that has been previously demonstrated to be effective in inhibiting microglia in the nanomolar–micromolar range [50, 84–87] or when they were depleted by 1-week treatment of PLX, which has been reported to induce a 90% reduction of IBA1^+^ cells in the hippocampal CA1 SR [51–54].

These results further support the role of microglia and of the CX3CL1–CX3CR1 axis in setting up the different basal levels of LTP observed in the two poles, strengthening the previous finding of an inverse relation between *Cx3cr1* level and LTP amplitude [37, 38]. Indeed, we found that the expression of *Cx3cr1* was higher in the VH where LTP is lower.

Overall, these observations suggest that microglia at steady-state conditions present a specific functional state in VH and DH, which is also indicated by the differences in their gene expression, morphology, and ultrastructure (see below). In particular, microglia in the DH appear to sustain higher hippocampal LTP level [88] since their inhibition/depletion or the *Cx3cr1* absence, reduces LTP. On the other hand, in VH, the same conditions enhance LTP amplitude, indicating a basal suppressive role of microglia in this pole. Further analysis is warranted to better elucidate mechanisms that underlie microglia-mediated alterations of hippocampal plasticity in VH and DH. One possibility is that the communication between neurons and microglia through the CX3CL1–CX3CR1 axis is needed to orchestrate gradients of several mediators, such as cytokines, neurotrophins, ATP/adenosine levels, that in turn could affect LTP amplitude differentially between the VH and DH. Microglia can impact synapse strength and plasticity through the release of cytokines under both physiological and pathological conditions [35, 89, 90] enhancing or suppressing LTP depending on their nature and concentration [35, 91], the sensitivity of different cell types to a given cytokine concentration, and the selective activation of receptor isoforms.

Cytokine network operating during LTP includes IL-1β, IL-6, and TNF-α [36, 92, 93]. As for IL-1β, besides its well-known inhibitory role in pathological conditions, emerging evidence also indicate an important physiological role for this cytokine in supporting hippocampus-dependent learning and plasticity [94–101]. Similar to IL-1β, TNF-α has been involved in synaptic plasticity and memory processes modulating post-synaptic α-amino-3-hydroxy-5-methyl-4-isoxazolepropionic acid receptor (AMPA) quantity [35, 89, 102, 103].

Further supporting the notion that microglial cytokines are relevant in setting the basal level of LTP, we observed lower levels of *Il-1β* and *Tnf-α* expression in CD11b^+^ cells from VH, where LTP amplitude is lower compared to the DH. It is important to note that the different expression of these cytokines between the DH and VH became evident when we separated microglia (CD11b^+^) from other brain cells. Given that both neurons and glia express cytokines, these results highlight the importance of isolating microglia when performing molecular analysis aimed to reveal the role of these cells in the healthy or damaged hippocampus.

Interestingly, some evidences also support a physiological role for IL-6 in synaptic plasticity [98, 104]. Microglia are important sources of IL-6 in the central nervous system [105], but also neurons can produce IL-6 under some conditions [106–110] while both an excess or a deficit of IL-6 can impact cognitive function. Studies indicate that IL-6 can serve as a negative regulator of both early and late LTP in the hippocampus [104, 111–113] through multiple signaling pathways [114]. In addition, this cytokine can strongly modulate astrocytes, which take part in the quadri-partite synapses and can also modulate synaptic plasticity [115]. Remarkably, IL-1β and IL-6 exert opposite effects on LTP [98, 104] and memory consolidation [116]. While treatment with IL-1β enhances AMPA or NMDA-mediated currents, IL-6 inhibits glutamate release and synaptic plasticity, and suppresses γ-aminobutyric acid (GABA)- and glycine-induced currents [113, 117]. We reported that expression of *Il-6* is enhanced while *Il-1β* is reduced in microglia isolated from VH, where LTP amplitude is lower compared to DH, further supporting the opposite action of these two cytokines in controlling basal LTP. Interestingly, the expression of *Bdnf*, a neurotrophin highly related to plasticity processes in the hippocampus [118–122], was augmented in whole DH and in CD11b^−^ cells magnetically separated from the DH region compared to VH, whereas it was similarly expressed by microglia in the two poles. These evidences imply that the observed different expression in total *Bdnf* mRNA depends on neuronal/astrocytic cells. Moreover, the higher expression of *Bdnf* was related to the high LTP measured in the DH, in line with the notion that this neurotrophin plays an essential role in hippocampal LTP modulation [120, 123].

Thus, it appears that proper chemokine signaling between neurons and microglia, particularly via the CX3CL1/CX3CR1 axis, and an adequate level of cytokines are essential for the maintenance of plasticity processes along the longitudinal axis under physiological conditions.

### Difference in microglial distribution, morphology, ultrastructure, and phagocytosis at the two poles

Previous research supports that microglia show marked hippocampal dorsoventral, interregional, and interlaminar differences [48, 124, 125]. To further investigate the diversity of microglia at steady-state, we assessed the density, distribution, morphology, and ultrastructure of IBA1^+^ cells in the CA1 SR of the two poles and found a significantly higher density of IBA1^+^ cells in the DH compared to the VH. Similarly, it has been observed that in the CA3 region of adult male rats, microglial density in the *strata oriens*, *radiatum* and *lacunosum-moleculare* was lower in the VH versus DH [48]. A higher density of IBA1^+^ cells in the DH could translate into increased surveillance of the hippocampal parenchyma and more functional microglia–neuron contacts, although future experiments would be necessary to elucidate this possibility. Our morphology analysis provides further insights into microglial heterogeneity at the two poles. Using a semi-automated approach, we found a significant increase in soma perimeter of IBA1^+^ cells in the CA1 SR, as well as in manual arborization perimeter, complemented by a reduction in arbor solidity and circularity in the VH versus DH, suggesting more cell ramifications in the ventral pole. Complementarily, by investigating the complexity of microglia shape through fractal analysis [59], we showed that IBA1^+^ cells have an increased lacunarity index in the VH compared to DH, indicative of a more complex or heterogeneous morphology within each cell. Similarly, in a male rat model of aseptic acute inflammation induced by neuraminidase, an increase of IBA1^+^ cell perimeter, fractal dimension, and lacunarity accompanied by a decrease in density was associated with more ramified morphologies [126]. Adaptations in microglial numbers, distribution and morphology are thought to assist these cells in effectively searching for microenvironmental cues in the brain parenchyma such as functional synaptic contacts [127]. We can speculate, that, in steady-state conditions, microglia in DH rely on increased density to perform their surveillance role, while microglia in the VH achieve similar scanning rates by adopting a larger, more ramified morphology.

Following this form-to-function paradigm, we used SEM to characterize microglial contacts and intracellular structures with the CA1 SR neuropil of the DH and VH [60]. The ultrastructural analysis of organelles showed that irrespective of the hippocampal pole, microglia display a uniform distribution of regular and dilated ER/Golgi apparatus, phagosomes, autophagosomes, and mitochondria. Furthermore, microglial cell body contacts with the neuropil were relatively similar between the DH and VH, with no significant changes found for the number of microglia interacting with neuronal cell bodies, myelinated axons, pre- and post-synaptic terminals, astrocytes, blood vessels, as well as extracellular space pockets (Additional file 1: Table S5).

We found that while microglia presenting immature lysosomes (primary and secondary lysosomes which are considered active) were more frequent in the VH, mature lysosomes (residual bodies named tertiary lysosomes, resulting from previous phagocytosis) were more prevalent in the DH. Our findings suggest that microglia present a higher phagocytic intake in the VH than in the DH pole. Mature lysosomes were previously associated with cellular aging and senescence in microglia, conditions where phagocytic intake and activity are also reduced [128–130]. However, the microglial lysosomal ability to degrade appears relatively unchanged between poles, as there were no differences in filled or empty phagosomes per animal and cell in the VH and DH. By contrast, in contexts of aging and neurodegenerative pathology, microglial lysosomes were found to display a reduced degradation capacity, associated with a higher pH [55, 129, 131]. During normal physiological conditions, ramified microglial processes can present ball-and-chain pouches that engulf apoptotic cells in the hippocampal subgranular zone of adult mice, a process important for neurogenesis [132]. Similarly, ball-and-chain structures are observed in ramified microglia phagocytosing synapses or extracellular debris after sensory experience in adolescent male mice [63], as well as during synaptic pruning in postnatal development [63, 133, 134]. Increased microglial ramification in the VH therefore does not exclude steady-state phagocytosis in the VH.

Remarkably, the expression of certain genes encoding recognized markers of microglial phagocytic activity was upregulated in the VH versus DH. In particular, *Mertk* and *Cd68* mRNA levels were increased in CD11b^+^ cells of the VH compared to the DH. *Mertk* mRNA expression was also increased in total RNA from the unsorted VH versus DH. In the adult mouse cortex of both sexes, *Mertk* was shown to coordinate astrocyte–microglial crosstalk for phagocytosis of dead neurons [135]. Additionally, mouse microglia upregulate their proliferation and lysosomal activity in cortical and hippocampal tissue, as assessed via CD68 protein expression, in response to increased mTOR signaling without associated inflammation [136]. Elevations in expression of phagocytic markers in the VH are aligned with the higher numbers of immature lysosomes detected by SEM, indicating overall increased rates of phagocytosis in the VH versus DH. However, more research is required to explore how this phagocytosis is executed by ramified cells and determine whether it involves the ball-and-chain mechanism [132]. Overall, to our knowledge, these results represent one of the first evidence of a region-specific modulation of microglia features at steady-state condition. Interestingly, it has been recently shown that under pathological conditions microglia can distinctively modulate gene expression between the two hippocampal poles. For instance, in an adult male rat model of hippocampal cholinergic deficit, the intracerebroventricular administration of Ig-saporin strongly upregulated gene expression of microglia-specific genes in the DH but not VH, as found by RNA-seq [137]. After chronic stress, an increase in the number of cells expressing the phagolysosomal activity marker CD68 was also found throughout layers of DG, CA1 and CA2 regions in the DH but was restricted to the DG of the VH [88].

### Basal functional state of microglia differs at the two hippocampal poles

Under basal conditions, microglia frequently display a small linear conductance while, following stimulation [138–140], these cells transiently express K_ir_ carried by K_ir_2.1 channel, and a K_or_ flowing through voltage-dependent K^+^ channels (K_v_) and Ca^2+^ activated K^+^ channels [68, 78, 140–143]. In primary rat and mouse microglia, the K_v_1.2, K_v_1.3, and K_v_1.5 mRNA transcripts and proteins have been detected, although at low levels [81, 144–147].

At present, the role of microglial K^+^ currents in physiological conditions is still poorly understood [47]. It is known that K_ir_ channels are specifically responsible for stabilization of the resting membrane potential and subsequent Ca^2+^ signaling [148, 149]. We reported similar amplitude of K_ir_ currents in DH and VH microglia and, in line with previous studies, the membrane potential values for microglial cells were comparable. By contrast, we found that microglia differ in their ability to produce K_or_ currents at the two hippocampal poles, being this current increased in VH compared to DH. Interestingly, this difference is associated with variations in *Il-1β*, *Tnf-α*, and *Il-6* expression levels in CD11b^+^ cells. These cytokines have been shown to be regulated by K^+^ channel, among CD11b^+^ cells. Specifically, K_v_1.3 seems to be a key factor in regulating the release of several cytokines upon stimuli and its knockout or deletion results in a reduction of IL-1β, TNF-α, and IL-6 release mediated by LPS [148, 150–152]. In addition, K_v_1.3 mRNA and channel expression were augmented by a pro-inflammatory stimuli such as LPS, IFN-γ, or TNF-α treatment [142, 145, 153, 154] or an anti-inflammatory one (IL-4) indicating that the elevated expression of this channel is not a reliable marker of exclusively pro-inflammatory microglial state [154]. Overall, these studies support the notion that the K_v_1.3 channels play a crucial role in controlling microglial release of cytokines. We speculate that in steady-state conditions, differences in this K^+^ current could be related to changes in *Il-1β*, *Tnf-α*, and *Il-6* expression. Although the percentage of microglia cells expressing K_or_ at both poles is around 50%, we can speculate that the different microglial K_or_ currents amplitude in VH versus DH could be linked to a specific level of cytokines released by microglia, that in turn could affect plasticity in a region-dependent manner. Further analyses are needed to better elucidate the mechanisms underlying this modulation and the functional implication of microglial differences in the K_or_ current at the two poles. Interestingly, the occurrence of the K_or_ current, which peaks during the second and third postnatal weeks, is reduced in *Cx3cr1*^*−/−*^ mice [155]. Here we observed that, in the Cx3cr1^+/GFP^ mice, the average amplitude of microglial outward K^+^ currents is increased in the VH, where *Cx3cr1* mRNA levels are higher compared to the DH, supporting a positive correlation between the CX3CR1 and K^+^ current occurrence. It must be stressed out that Cx3cr1^+/GFP^ mice*,* extensively used to characterize microglia features, cannot be considered as wild-type mice, as they show an intermediate phenotype between wild-type and Cx3cr1^−/−^ in different parameters such as LTP basal amplitude [37]. Therefore, it is possible that using Cx3cr1^+/GFP^ mice we underestimate the differences in K^+^ currents, and further analysis in CTRL mice will be necessary to better elucidate microglia functional state in DH and VH.

### Future perspectives

Growing evidence suggest that microglia are sexually dimorphic in the healthy and diseased brain [156–158]. In particular, during different stages of the postnatal brain development and in the adult, gender is an important determinant of expression patterns, numbers and morphology of microglia in several brain regions [159–161]. In addition, following injury, inflammation and stress, marked sex differences in the microglial activation patterns emerge [156, 162, 163]. Taken together, this evidence highlights the importance of analyzing microglia features and function in both females and males. In the present paper, we elucidated microglial diversity along the hippocampal longitudinal axis in males, but further experiments are warranted to better explore the mechanisms underlying these differences at the two poles and to clarify possible sex differences in this context.

## Conclusions

In conclusion, microglia show regional density, morphological, ultrastructural, and physiological adaptations at the different hippocampal poles, contributing to differences in regional hippocampal activity. Importantly, CX3CL1–CX3CR1 signaling is critical in setting the level of LTP of DH and VH under basal conditions, possibly orchestrating the environmental concentration of several mediators, including *Bdnf*, *Il-1β*, *Tnf-α*, and *Il-6*, of both glial and neuronal origins.

All the presented findings underscore the importance of considering dorsal and ventral hippocampus separately and microglia as important factors of this functional segregation, which has relevant implications for fundamental research and important clinical implications. This approach would facilitate a better understanding of how changes in these subregions induced by different microglial statuses can contribute to the development and treatment of specific phenotypical aspects of several diseases in which microglia are involved.

## Supplementary Information

**Additional file 1: Table S1.** Primers sequence for real-time polymerase chain reaction experiments. **Table S2.** Mean of the CT values for target and housekeeping genes obtained in total mRNA extracted from the whole DH and VH, in CD11b^+^ cells, and CD11b^−^ cells for CD11b^+^, CD11b^−^ by real-time polymerase chain reaction experiments. **Table S3.** Density and distribution parameters of IBA1^+^ cells in the dorsal versus ventral hippocampus CA1 *stratum radiatum*. **Table S4.** Light microscopy parameters of IBA1^+^ cells in the dorsal versus ventral hippocampus CA1 *stratum radiatum*. **Table S5.** Electron microscopy parameters of IBA1^+^ cells in the dorsal versus ventral hippocampus CA1 *stratum radiatum*.

## Data Availability

All data are available in the main text or the Additional file. The data that support the findings of this study are available from the corresponding author upon reasonable request.

## Abbreviations

LTP: Long-term potentiation
DH: Dorsal hippocampus
VH: Ventral hippocampus
CA: *Cornu Ammonis*
NMDAR: *N*-Methyl-d-aspartate receptor
IL: Interleukin
TNF: Tumor necrosis factor
BDNF: Brain derived neurotrophic factor
CX3CL1: Fractalkine
CX3CR1: Fractalkine receptor
CTRL: C57BL/6J wild-type
GFP: Green fluorescent protein
MINO: Minocycline
PLX: PLX5622
ACSF: Artificial cerebrospinal fluid
SR: *Stratum radiatum*
fEPSP: Field excitatory post-synaptic potentials
HFS: High-frequency stimulation
PPR: Paired pulse ratio
K_or_: Outward rectifier potassium current
K_ir_: Inward rectifier potassium currents
SEM: Scanning electron microscopy
IBA1: Ionized calcium-binding adapter molecule 1
ROI: Region of interest
NND: Nearest neighbor distance
a.u.: Arbitrary unit
ER: Endoplasmic reticulum
CD: Cluster of differentiation
MACS: Magnetic-activated cell sorting
rt-PCR: Real time polymerase chain reaction
CypA: Cyclophilin A
GAPDH: Glyceraldehyde 3-phosphate dehydrogenase
S.E.M.: Standard error of mean
Mertk: Mer proto-oncogene tyrosine kinase
Trem2: Triggering receptor expressed on myeloid cells 2
GABA: G-aminobutyric acid
K_v_: Voltage-dependent K^+^ channels
