# Supplementary material for: Microglial diversity along the hippocampal longitudinal axis impacts synaptic plasticity in adult male mice under homeostatic conditions
Source: J Neuroinflammation. 2022 Dec 8;19:292. doi: 10.1186/s12974-022-02655-z (PMC9730634; doi:10.1186/s12974-022-02655-z)
Supplement: Supplementary file 1 — Additional file 1: Table S1. Primers sequence for real-time polymerase chain reaction experiments. Table S2. Mean of the CT values for target and housekeeping genes obtained in total mRNA extracted from the whole DH and VH, in CD11b+ cells, and CD11b− cells for CD11b+, CD11b− by real-time polymerase chain reaction experiments. Table S3. Density and distribution parameters of IBA1+ cells in the dorsal versus ventral hippocampus CA1 stratum radiatum. Table S4. Light microscopy parameters of IBA1+ cells in the dorsal versus ventral hippocampus CA1 stratum radiatum. Table S5. Electron microscopy parameters of IBA1+ cells in the dorsal versus ventral hippocampus CA1 stratum radiatum. [file 12974_2022_2655_MOESM1_ESM.docx]

**Table S1. Primers sequence for real-time polymerase chain reaction experiments.**

| Gene | Primer Forward (5’ – 3’) | Primer Reverse (3’ – 5’) |
| --- | --- | --- |
| CypA | AGCATACAGGTCCTGGCATC | TTCACCTTCCCAAAGACCAC |
| Gapdh | TCGTCCCGTAGACAAAATGG | TTGAGGTCAATGAAGGGGTC |
| Cx3cr1 | TGACTGGCACTTCCTGCAGA | AGGGCGTAGAAGACGGACAG |
| Cx3cl1 | CTTCCATTTGTGTACTCTGCTGC | GACTCCTGGTTTAGCTGATAGCG |
| IL-1β | TGAAAGCTCTCCACCTCAATG | CCAAGGCCACAGGTATTTTG |
| TNF-α | GGCCTCCCTCTCATCAGTTC | CACTTGGTGGTTTGCTACGA |
| IL-6 | CTTCACAAGTCGGAGGCTTA | CAAGTGCATCATCGTTGTTC |
| BDNF | TCGTTCCTTTCGAGTTAGCC | TTGGTAAACGGCACAAAAC |
| Trem2 | GATTCTGGCCAGCACAACAGA | GAGATATCCGGTAGCCCACCA |
| Mertk | CTTATGACGCCTTGAAGCACTG | CACCGGCTTGGAGGTTCTTC |
| CD68 | CTTATGGACAGCTTACCTTT | AGCTCTCGAAGAGATGAAT |

**Table S2. Mean of the CT values for target and housekeeping genes obtained in total mRNA extracted from the whole DH and VH, in CD11b^+^ cells, and CD11b^-^ cells for CD11b^+^, CD11b^-^ by real-time polymerase chain reaction experiments.**

| **Gene** | **Brain area** | **Sample** | **Mean Ct Target** | **Mean Housekeeping** |
| --- | --- | --- | --- | --- |
| Cx3cr1 | DH | CD11b+ | 25.55 | 24.32 |
| Cx3cr1 | VH | CD11b+ | 25.70 | 25.15 |
| Cx3cl1 | DH | CD11b- | 27.57 | 24.32 |
| Cx3cl1 | VH | CD11b- | 29.08 | 25.74 |
| IL-1β | DH | Whole | 32.83 | 19.17 |
| IL-1β | VH | Whole | 33.13 | 19.35 |
| TNF-α | DH | Whole | 33.02 | 19.17 |
| TNF-α | VH | Whole | 33.21 | 19.35 |
| IL-6 | DH | Whole | 33.46 | 19.17 |
| IL-6 | VH | Whole | 33.95 | 19.35 |
| BDNF | DH | Whole | 25.05 | 19.17 |
| BDNF | VH | Whole | 25.99 | 19.35 |
| IL-1β | DH | CD11b+ | 36.16 | 27.38 |
| IL-1β | VH | CD11b+ | 36.63 | 26.54 |
| TNF-α | DH | CD11b+ | 27.42 | 19.02 |
| TNF-α | VH | CD11b+ | 31.11 | 20.69 |
| IL-6 | DH | CD11b+ | 32.04 | 23.20 |
| IL-6 | VH | CD11b+ | 31.83 | 22.35 |
| BDNF | DH | CD11b+ | 33.33 | 23.20 |
| BDNF | VH | CD11b+ | 33.25 | 22.35 |
| IL-1β | DH | CD11b- | 36.59 | 24.32 |
| IL-1β | VH | CD11b- | 37.26 | 25.74 |
| TNF-α | DH | CD11b- | 32.71 | 24.32 |
| TNF-α | VH | CD11b- | 33.77 | 25.74 |
| IL-6 | DH | CD11b- | 34.17 | 24.32 |
| IL-6 | VH | CD11b- | 34.89 | 25.74 |
| BDNF | DH | CD11b- | 31.28 | 24.32 |
| BDNF | VH | CD11b- | 33.64 | 25.74 |
| Trem2 | DH | Whole | 26.12 | 19.17 |
| Trem2 | VH | Whole | 26.25 | 19.35 |
| Merk | DH | Whole | 25.01 | 19.17 |
| Merk | VH | Whole | 24.66 | 19.35 |
| Cd68 | DH | Whole | 26.30 | 19.17 |
| Cd68 | VH | Whole | 26.35 | 19.35 |
| Trem2 | DH | CD11b+ | 27.70 | 27.38 |
| Trem2 | VH | CD11b+ | 27.61 | 26.54 |
| Merk | DH | CD11b+ | 29.18 | 23.20 |
| Merk | VH | CD11b+ | 25.98 | 22.35 |
| Cd68 | DH | CD11b+ | 26.90 | 23.20 |
| Cd68 | VH | CD11b+ | 24.95 | 22.35 |

**Table S3.** **Density and distribution parameters of IBA1^+^ cells in the dorsal *versus* ventral hippocampus CA1 *stratum radiatum.***

| **Parameters** | **Dorsal** | | **Ventral** | |
| --- | --- | --- | --- | --- |
|  | Mean | S.E.M. | Mean | S.E.M. |
| **Density** (cells/mm^2^) | 436.8 | 14.29 | 321.2 | 14.69 |
| **NND** (µm) | 34.76 | 0.5373 | 38.48 | 0.5255 |
| **Spacing index** (arbitrary units) | 0.5212 | 0.0064 | 0.4728 | 0.0121 |

Each parameter was averaged per animal for statistical analysis. For each data, Mean and S.E.M of n=6 animals are given. p-value are given after paired, parametric Student’s t-test. S.E.M. standard error of the mean

**Table S4. Light microscopy parameters of IBA1^+^ cells in the dorsal *versus* ventral hippocampus CA1 *stratum radiatum.***

| **Parameters** | | **Dorsal** | | | | **Ventral** | | | | p-value |
| --- | --- | --- | --- | --- | --- | --- | --- | --- | --- | --- |
|  |  | Mean | S.E.M | Min | Max | Mean | S.E.M | Min | Max |  |
| **Manual Soma** | Area  (μm^2^) | 36.873 | 0.957 | 34.365 | 40.576 | 38.474 | 0.870 | 35.789 | 41.276 | 0.086 |
|  | Perimeter (μm) | 27.050 | 0.470 | 25.527 | 28.242 | 28.298 | 0.631 | 26.602 | 30.674 | 0.023* |
| **Manual arbor** | Area  (μm^2^) | 1523.021 | 102.454 | 1277.014 | 1966.489 | 1716.295 | 45.520 | 1599.958 | 1893.369 | 0.094 |
|  | Perimeter  (μm) | 263.482 | 11.433 | 236.309 | 306.134 | 291.202 | 7.796 | 265.959 | 311.459 | 0.010* |
|  | Circularity (a.u.) | 0.295 | 0.010 | 0.271 | 0.337 | 0.271 | 0.006 | 0.246 | 0.287 | 0.024* |
|  | Aspect ratio (a.u.) | 1.590 | 0.062 | 1.363 | 1.755 | 1.588 | 0.058 | 1.397 | 1.792 | 0.986 |
|  | Roundness (a.u.) | 0.665 | 0.021 | 0.610 | 0.752 | 0.661 | 0.021 | 0.587 | 0.741 | 0.917 |
|  | Solidity (a.u.) | 0.720 | 0.007 | 0.700 | 0.742 | 0.703 | 0.004 | 0.688 | 0.724 | 0.051 |
| **Automatic arbor** | Area (μm^2^) | 418.634 | 36.916 | 335.086 | 571.854 | 446.093 | 18.577 | 405.218 | 531.056 | 0.414 |
|  | Perimeter (μm) | 658.325 | 44.679 | 559.836 | 860.572 | 724.746 | 21.425 | 658.578 | 811.875 | 0.152 |
|  | Circularity (a.u.) | 0.016 | 0.003 | 0.010 | 0.032 | 0.011 | 0.0003 | 0.010 | 0.013 | 0.093 |
|  | Aspect ratio (a.u.) | 1.635 | 0.073 | 1.379 | 1.845 | 1.626 | 0.056 | 1.471 | 1.831 | 0.935 |
|  | Roundness (a.u.) | 0.652 | 0.023 | 0.583 | 0.741 | 0.649 | 0.019 | 0.577 | 0.705 | 0.916 |
|  | Solidity (a.u.) | 0.218 | 0.008 | 0.198 | 0.250 | 0.195 | 0.004 | 0.192 | 0.223 | 0.031* |
|  | Branches (number) | 117.300 | 11.190 | 97.000 | 167.000 | 129.200 | 8.179 | 111.000 | 167.000 | 0.343 |
|  | Junctions (number) | 58.830 | 5.659 | 48.000 | 84.000 | 64.500 | 4.233 | 55.000 | 84.000 | 0.316 |
|  | Max branch length (μm) | 16.765 | 0.466 | 15.091 | 18.251 | 17.047 | 0.285 | 16.401 | 18.082 | 0.709 |
|  | Longest shortest path branch (μm) | 94.424 | 2.519 | 88.915 | 105.429 | 100.244 | 1.991 | 94.259 | 107.492 | 0.128 |
|  | Fractal dimension (a.u.) | 1.441 | 0.011 | 1.413 | 1.485 | 1.443 | 0.007 | 1.428 | 1.481 | 0.437 |
|  | Lacunarity (a.u.) | 0.398 | 0.004 | 0.377 | 0.412 | 0.418 | 0.005 | 0.397 | 0.435 | 0.002** |

Each parameter was analyzed per microglial cell and averaged per animal for statistical analysis. For each data, Mean ± S.E.M, Min, Max of n=6 animals are given. p-value are given after paired, two-tailed analyses (Student’s t-test for parametric and Wilcoxon test for nonparametric datasets). p-value of significant statistical tests are followed by an asterisk indicating the significance, * < 0.05, ** < 0.01. a.u. arbitrary units, Min minimum, Max maximum, S.E.M. standard error of the mean.

**Table S5. Electron microscopy parameters of IBA1^+^ cells in the dorsal *versus* ventral hippocampus CA1 *stratum radiatum.***

| **Parameters** | | Data | **Dorsal** | | | | **Ventral** | | | | p-value |
| --- | --- | --- | --- | --- | --- | --- | --- | --- | --- | --- | --- |
|  |  |  | Mean | S.E.M. | Min | Max | Mean | S.E.M. | Min | Max |  |
| **Organelles** | **Empty  phagosomes** | # | 0.354 | 0.126 | 0.000 | 0.600 | 0.633 | 0.314 | 0.083 | 1.250 | 0.333 |
|  |  | % | 0.283 | 0.104 | 0.000 | 0.500 | 0.345 | 0.152 | 0.083 | 0.700 | 0.665 |
|  | **Filled  phagosomes** | # | 0.341 | 0.137 | 0.000 | 0.666 | 0.372 | 0.254 | 0.000 | 1.125 | 0.927 |
|  |  | % | 0.275 | 0.110 | 0.000 | 0.500 | 0.222 | 0.138 | 0.275 | 0.110 | 0.821 |
|  | **Autophagosomes** | # | 0.133 | 0.081 | 0.000 | 0.333 | 0.112 | 0.065 | 0.000 | 0.250 | 0.864 |
|  |  | % | 0.087 | 0.059 | 0.000 | 0.250 | 0.112 | 0.065 | 0.000 | 0.250 | 0.820 |
|  | **Phagosomes** | # | 0.695 | 0.239 | 0.000 | 1.083 | 1.006 | 0.528 | 0.100 | 2.375 | 0.582 |
|  |  | % | 0.441 | 0.151 | 0.000 | 0.666 | 0.4604 | 0.1927 | 0.100 | 0.875 | 0.930 |
|  | **Immature  lysosomes** | # | 0.791 | 0.219 | 0.250 | 1.300 | 1.052 | 0.1704 | 0.583 | 1.400 | 0.246 |
|  |  | % | 0.350 | 0.061 | 0.250 | 0.500 | 0.489 | 0.059 | 0.333 | 0.625 | 0.034* |
|  | **Mature  lysosomes** | # | 0.254 | 0.051 | 0.166 | 0.400 | 0.191 | 0.108 | 0.000 | 0.500 | 0.386 |
|  |  | % | 0.229 | 0.064 | 0.100 | 0.400 | 0.160 | 0.079 | 0.000 | 0.375 | 0.023* |
|  | **Elongated mitochondria** | # | 0.350 | 0.095 | 0.100 | 0.500 | 0.133 | 0.056 | 0.000 | 0.250 | 0.243 |
|  |  | % | 0.220 | 0.048 | 0.100 | 0.333 | 0.133 | 0.056 | 0.000 | 0.250 | 0.463 |
|  | **Mitochondria  with vacuoles** | # | 0.212 | 0.108 | 0.000 | 0.500 | 0.125 | 0.125 | 0.000 | 0.500 | 0.750 |
|  |  | % | 0.212 | 0.108 | 0.000 | 0.500 | 0.093 | 0.093 | 0.000 | 0.375 | 0.750 |
|  | **Dilated endoplasmic reticulum/Golgi** | # | 0.856 | 0.154 | 0.500 | 1.250 | 1.075 | 0.428 | 0.250 | 2.250 | 0.689 |
|  |  | % | 0.531 | 0.051 | 0.400 | 0.625 | 0.510 | 0.150 | 0.166 | 0.875 | 0.908 |
| **Interactions** | **Extracellular**  **digestion** | # | 0.145 | 0.065 | 0.000 | 0.300 | 0.168 | 0.098 | 0.000 | 0.375 | 0.701 |
|  |  | % | 0.095 | 0.041 | 0.000 | 0.200 | 0.118 | 0.088 | 0.000 | 0.375 | 0.808 |
|  | **Astrocyte  cell body** | # | 0.433 | 0.070 | 0.300 | 0.600 | 0.356 | 0.154 | 0.000 | 0.750 | 0.592 |
|  |  | % | 0.204 | 0.044 | 0.100 | 0.300 | 0.185 | 0.087 | 0.000 | 0.416 | 0.846 |
|  | **Blood  vessel** | # | 0.225 | 0.043 | 0.100 | 0.300 | 0.241 | 0.066 | 0.100 | 0.416 | 0.831 |
|  |  | % | 0.225 | 0.0433 | 0.100 | 0.300 | 0.2417 | 0.0661 | 0.100 | 0.416 | 0.831 |
|  | **Neuronal  cell body** | # | 0.050 | 0.050 | 0.000 | 0.200 | 0.127 | 0.025 | 0.083 | 0.200 | 0.250 |
|  |  | % | 0.050 | 0.050 | 0.000 | 0.200 | 0.1271 | 0.0257 | 0.083 | 0.200 | 0.250 |
|  | **Post-synaptic  element** | # | 2.115 | 0.502 | 0.875 | 3.200 | 1.558 | 0.391 | 0.833 | 2.500 | 0.243 |
|  |  | % | 0.779 | 0.096 | 0.500 | 0.916 | 0.708 | 0.074 | 0.583 | 0.900 | 0.529 |
|  | **Pre-synaptic  element** | # | 7.085 | 2.193 | 3.625 | 13.300 | 8.100 | 2.398 | 3.900 | 12.500 | >0.999 |
|  | **Myelinated  axon** | # | 0.147 | 0.022 | 0.100 | 0.200 | 0.077 | 0.027 | 0.000 | 0.125 | 0.139 |
|  |  | % | 0.147 | 0.022 | 0.100 | 0.200 | 0.077 | 0.027 | 0.000 | 0.125 | 0.139 |
|  | **Extracellular  space** | # | 1.060 | 0.3871 | 0.125 | 2.000 | 1.196 | 0.130 | 0.900 | 1.500 | 0.685 |
|  |  | % | 0.535 | 0.163 | 0.125 | 0.800 | 0.550 | 0.028 | 0.500 | 0.600 | >0.999 |

Each parameter was analyzed per microglial cell body and averaged per animal for statistical analysis. For each data, Mean ± S.E.M., Min, Max of n=4 animals are given. The symbol # represents the mean absolute value per animal, whereas % represents the mean relative percentage of cells per animal. All microglia analyzed made direct interactions with pre-synaptic elements, therefore the corresponding relative values obtained were not included in the Table. p-values are given after paired, two-tailed analyses (Student’s t-test for parametric and Wilcoxon test for nonparametric datasets). p-values of significant statistical tests are followed by an asterisk indicating the significance, * < 0.05, ** < 0.01. a.u. arbitrary units, Min minimum, Max maximum, S.E.M. standard error of the mean.
